# Effect of Alkyl Side Chain Length on the Lithium-Ion Conductivity for Polyether Electrolytes

**DOI:** 10.3389/fchem.2022.943224

**Published:** 2022-07-14

**Authors:** Ryansu Sai, Seiko Hirata, Hiromori Tsutsumi, Yu Katayama

**Affiliations:** ^1^ Department of Applied Chemistry, Graduate School of Sciences and Technology for Innovation, Yamaguchi University, Ube, Japan; ^2^ Department of Energy and Environmental Materials, SANKEN, Osaka University, Ibaraki, Japan

**Keywords:** lithium-ion battery (LIB), polymer electrolytes, ether group, ion transport, alkyl side chain

## Abstract

The design guidelines of polymer structure to effectively promote lithium-ion conduction within the polymer electrolytes (PEs) are crucial for its practical use. In this study, the electrolyte properties of a simple polyether having alkyl side chains with varied lengths (−(CH_2_)_m_−H, *m* = 1, 2, 4, 6, 8, and 12) were compared and established a valid design strategy based on the properties of the alkyl side chain. Various spectro-electrochemical measurements successfully connected the electrolyte properties and the alkyl side chain length. Steric hindrance of the alkyl side chain effectively suppressed the interaction between ether oxygen and lithium-ion (*m* ≥ 2), decreasing the glass transition temperature and the activation energy of lithium-ion transfer at the electrode-electrolyte interface. The strong hydrophobic interactions aligned and/or aggregated the extended alkyl group (*m* ≥ 8), creating a rapid lithium-ion transport pathway and enhancing lithium-ion conductivity. A clear trend was observed for the following three crucial factors determining bulk lithium-ion transport properties along with the extension of the alkyl side chain: 1) salt dissociability decreased due to the non-polarity of the alkyl side chain, 2) segmental mobility of polymer chains increased due to the internal plasticizing effect, and 3) lithium-ion transference number increased due to the inhibition of the bulky anion transport by its steric hindrance. The highest lithium-ion conductivity was confirmed for the PEs with an alkyl side chain of moderate length (*m* = 4) at 70°C, indicating the optimized balance between salt dissociability, polymer segmental mobility, and selective lithium-ion transfer. The length of an alkyl side chain can thus be a critical factor in improving the performance of PEs, including thermal stability and lithium-ion conductivity. Precise tuning of the alkyl side chain-related parameters such as steric hindrance, polarity, internal plasticizing effect, and self-alignment optimizes the polymer segmental mobility and salt dissociability, which is crucial for realizing high lithium-ion conductivity for PEs.

## Introduction

Polymer electrolytes (PEs), containing lithium-conducting polymers and lithium salts, are considered a vital element of the next-generation secondary batteries, enabling the use of highly active lithium-metal electrodes due to their high thermal and mechanical stability (Zhou et al., 2019). High thermal stability of polymers (e.g., the flashpoint of poly(ethylene glycol) is 254°C, which is higher than that of short molecules with similar structures like 1,2-dimethoxyethane (−6°C) or triethylene glycol dimethyl ether (111°C)) (Tang and Zhao, 2014) may suppress the thermal/electrochemical decomposition of the electrolytes on a lithium-metal electrode (Wang et al., 2012), leading to improve the safety and cycle characteristics of the batteries (An et al., 2016). Furthermore, the high mechanical stability of PEs suppresses the growth of lithium-metal dendrites (Monroe and Newman, 2005; Bae et al., 2019), which is the cause of capacity loss (Fang et al., 2019) and short circuit (Feng et al., 2018) of batteries. Despite those advantages, low ionic conductivity is one of the crucial problems for PEs. Liquid electrolyte used in general, e.g., 1 M LiPF_6_ in ethylene carbonate/diethyl carbonate (1/1, v/v), showed ionic conductivity of 8 × 10^–3^ S cm^−1^at room temperature. (Lundgren et al., 2014). In contrast, the ionic conductivity of the standard PEs, poly(ethylene oxide) (PEO) with lithium salts, is only ≤ 1 × 10^−5^ S cm^−1^ (Fergus, 2010). The low ionic conductivity of PEs causes large overvoltage and large Joule heat, which can be the reason for the energy loss and thermal runaway of the batteries, respectively (Wang et al., 2012). Although various polymers have been proposed for electrolyte matrix since the 1970s, (Wright, 1975), the practicable ionic conductivity (≥ 1 × 10^−3^ S cm^−1^) has not yet been achieved.

The ionic conductivity of the PEs is primarily determined by the ion mobility and the number of ion carriers. Lithium-ion within the PEs has been reported to be transferred *via* two mechanisms (Diddens et al., 2010; Brooks et al., 2018): (I) polymer chain carries lithium-ion with its segmental motion while maintaining the Li^+^-coordination structure (which can be rephrased to Li^+^-solvation structure for liquid electrolytes) and/or (II) exchange from the one coordination site to another during the reorganization of Li^+^-coordination structure. Therefore, the coordination structure around lithium-ion, more specifically, the stability of the Li^+^-coordination structure, can be one of the design descriptors for improving lithium-ion transport within the PEs; the moderately stable Li^+^-coordination structure promotes salt dissociation (Shirota et al., 2016) while too stable Li^+^-coordination structure raises the energy barrier of the structural reorganization required for smooth lithium-ion transport (Webb et al., 2015). Furthermore, a highly stable Li^+^-coordination structure reduces the segmental mobility of the polymer chain by inhibiting the polymer bond rotation (Siqueira and Ribeiro, 2005). Although optimizing the coordination structure is necessary for improving the lithium-ion transport property for the PEs, the effective strategy for controlling the coordination structure is not well established despite our previous approaches (Sai et al., 2017a; Sai et al., 2017b; Matsuoka et al., 2020; Yamada et al., 2021). One of the reasons is the significant structural difference between conventional solvent and polymer systems; all the molecules can move individually in the solution system while the alkyl backbone connects each polar group in the polymer system, limiting the possible coordination structure.

Designing the steric effect of the alkyl chain is one of the potential strategies to control the coordination structure within the PEs. Redfern and Curtiss (2002) compared the coordination structures of PEO and poly (tetramethylene oxide) (PTMO) and clarified that steric crowding around lithium-ion for PTMO electrolyte decreased the stability of the coordination structure. However, changing the steric effect of the alkyl chain also affects physicochemical properties, including dielectric constant, internal free volume, and hydrophobicity, which affect the salt dissociability (Wheatle et al., 2017), polymer segmental mobility (Mauritz et al., 1990), and lithium-ion conduction pathway (Liu et al., 2005; Liu et al., 2006), respectively. Therefore, to establish the polymer design strategy based on the fine-tuning of the alkyl chain length, it is essential to clarify the effect of the alkyl chain on the various physicochemical properties, including the coordination structure.

**FIGURE 1:**
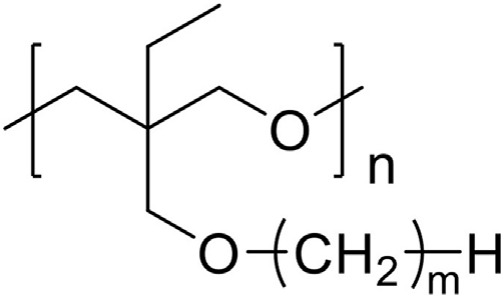
Structure of the matrix polymer, PCmEO (*m* = 1, 2, 4, 6, 8, and 12).

In this study, we investigated the influence of the alkyl side chain on the coordination structure around lithium-ion and ether groups, as well as the lithium-ion transport properties for polyether electrolytes. We systematically compared model polymers with varied alkyl side chain length, poly [3-ethyl-3-(*alkyl*)oxymethyloxetane] (alkyl; methyl, ethyl, butyl, hexyl, octyl, and dodecyl, described as PCmEO, *m* = 1, 2, 4, 6, 8, and 12) (Figure 1), to establish a simple and effective polymer design strategy based on the tuning of the alkyl side chain. The length of the alkyl side chain affects the stability of the lithium coordination structure; the electrolyte with a short alkyl side chain (*m* = 1) formed the stable coordination structure due to the multiple interactions between ether groups and lithium-ion. Relatively large steric hindrance of the alkyl side chain (*m* = 2−12) effectively suppressed the interaction between ether oxygen and lithium-ion, which slightly destabilized the lithium coordination structure and improved the segmental mobility as well as the activation energy of lithium-ion transfer at the electrode-electrolyte interface. Polymer segmental mobility was also improved with the extension of the alkyl side chain due to the internal plasticizing effect. The dielectric constant of the polymers decreased by extending the non-polar alkyl side chain, reducing the salt dissociability. Lithium-ion transference number increased with the extension of the alkyl side chain, indicating the effective suppression of the bulky anion transfer by its steric hindrance. In addition, the space between well-aligned alkyl side chains (*m* = 12) can act as a rapid lithium-ion pathway, leading to the high lithium-ion conductivity of 2.94 × 10^−6^ S cm^−1^ at 70°C. The results highlight that the alkyl side-chain length dictates critical factors determining lithium-ion conductivity, such as lithium transfer energetics both in bulk and at the electrode-electrolyte interface, polymer segmental mobility, and salt dissociability. The precise tuning of an alkyl side chain can thus be a simple and effective design strategy to improve the PE properties required for realizing safe and high-performance all-solid rechargeable battery technologies.

## Experimental

### Preparation of Polymer Electrolyte

**SCHEME 1:**
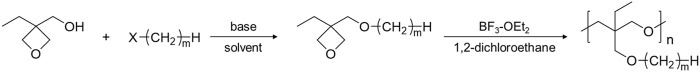
Synthesis scheme of CmEO and PCmEO (*m* = 1, 2, 4, 6, 8, and 12). X stands for the following leaving groups: iodo, bromo, or methanesulfonyl.

Poly (3-ethyl-3-(*alkyl*)oxymethyloxetane), PCmEO, having an alkyl side chain with various lengths (*m* = 1, 2, 4, 6, 8, and 12) was used in this study (Figure 1). PCmEO was synthesized based on previous studies (Mu et al., 2013; Sai et al., 2017a). The monomers of PCmEO and CmEO were synthesized from the coupling of 3-ethyl-3-hydroxymethyloxetane (EHO) and alkyl compounds modified with leaving groups in the base solution (Scheme 1). Prepared 3-ethyl-3-(*alkyl*)oxymethyloxetane (CmEO) was polymerized by ring-opening cationic polymerization (ROCP) using the cationic initiator, BF_3_-OEt_2_, in the solvent of 1,2-dichloroethane. Synthesis details are described in Supporting Information. The successful synthesis of PCmEO (*m* = 1, 2, 4, 6, 8, and 12) was confirmed by ^1^H NMR spectrometry (Supplementary Information). The molecular weight of each PCmEO obtained from the chromatogram of gel permeation chromatography (GPC) (Supplementary Figure S3; Supplementary Table S1) was more than 10 kg mol^−1^, which was large enough to exclude the contribution of the molecular weight of the polymer on the physicochemical properties (Teran et al., 2011). PCmEO electrolytes were prepared by mixing the PCmEO and 1.0 M lithium bis(trifluoromethanesulfonyl)amide (LiTFSA) in tetrahydrofuran (THF) solution, subsequently heated under a vacuum to remove THF, which was confirmed using infrared spectroscopy. The mixing ratio of polymer repeating unit and LiTFSA was set to 5. Prepared PEs are denoted as PCmEO-LiTFSA throughout the study. The amount of LiTFSA in PEs, LiTFSA weight ratio, and LiTFSA concentration are listed in Supplementary Table S2.

### Characterization Methods

The structures of the synthesized compounds were identified using nuclear magnetic resonance (NMR) spectroscopy. The 1H NMR and 13C NMR spectra were recorded with an FT-NMR spectrophotometer (JNM-LA-500, JEOL). The molecular weight of the polymers was measured by a gel permeation chromatography (GPC) system (CBM-20A, LC-20AD, DGU-20A, CTO-20A, and RID-10A, Shimadzu) using THF as the elution solvent and polystyrene standards for the calibration of columns.

A differential thermogravimetric analyzer (Thermo Plus Evo II, RIGAKU) was used to determine the thermal decomposition temperature (*T*_d_). A small amount of the pure polymers or PEs (∼10 mg) was put on an open aluminum pan. Pure polymer samples were heated from room temperature to 500°C at a heating rate of 20°C min^−1^. Polymer electrolytes were first heated to 150°C and kept for 10 min to remove absorbed water and then cooled to 50°C and then heated again to 500°C at a heating rate of 20°C min^−1^. The final heating scan was recorded as the thermogravimetry (TG) curve of the samples. The measurements were performed under He flow. Thermal decomposition temperature was determined as the intersection of the baseline and the diagonal line of the most significant mass loss. Gas chromatography–mass spectrometry (GC-MS) was performed by a gas chromatography/mass spectrometer (GCMS-QP2010 Ultra, SHIMADZU) with a double-shot pyrolyzer (PY-2020iD, Frontier Lab) and thermal desorption system (TD-20, SHIMADZU). A small amount of PC1EO-LiTFSA or PC12EO-LiTFSA (∼0.6 mg) and quarts of wool were put into a stainless-steel cup. The sample was first heated to 150°C and kept for 10 min, then heated again to 500°C at a heating rate of 20°C min^−1^.

A differential scanning calorimeter (DSC7020, HITACHI) was used to determine the glass transition temperature (*T*_g_), re-crystallization temperature (*T*_c_), and melting temperature (*T*_m_). A small amount of the polymers or the PEs (∼10 mg) was hermetically sealed in an aluminum DSC pan. The samples were first heated to 120°C and cooled to −100°C, and then heated again to 120°C at a heating/cooling rate of 10°C min^−1^ (for PC12EO and PC12EO-LiTFSA, the samples were cooled to −150°C). The second heating scan was recorded as the DSC thermogram of the samples. The thermal transition temperatures (*T*_g_, *T*_c_, and *T*_m_) were determined as the intersection of the baseline and the diagonal lines of the second heat flow step.

Infrared (IR) spectra of the PEs were measured by a Fourier transform infrared spectrometer (Nicolet iS50, Thermo-Fisher Scientific) in the wavenumber range of 550−4000 cm^−1^ with a resolution of 4 cm^−1^. The measurements were performed by attenuated total reflection (ATR) configuration with a ZnSe prism. The polymer electrolyte was sealed between the ATR stage and glass plate with the grease in an Ar-filled glove box to avoid moisture absorption. The Raman spectra of the PEs were measured by a laser Raman spectrophotometer (MRS-3100, JASCO) equipped with a 532-nm laser. The polymer electrolyte was sealed between two glass plates having a rubber spacer (5 mm in thickness) in an Ar-filled glove box to avoid moisture absorption. Raman spectra were measured in the wavenumber range of 100−3000 cm^−1^ with a resolution of 4 cm^−1^. The spectra of IR and Raman were analyzed with commercially available peak fitting software (PeakFit™ ver. 4.12, SeaSolve Co.). A pseudo-Voigt function was used for the peak deconvolution.

### Electrochemical Measurements

Linear sweep voltammetry (LSV) and cyclic voltammetry (CV) were performed to study the electrochemical stability of the PEs. Each PCmEO-LiTFSA infiltrated in glass filter paper (GA-55, ADVANTEC) was placed between electrodes in a 2032-type cell. Linear sweep voltammograms were obtained using lithium-metal foil as the counter and reference electrode and a stainless-steel disk as the working electrode. Cyclic voltammograms were obtained using lithium-metal foil as the counter and reference electrodes and a nickel disk as the working electrode. The cell was stabilized by storing it in a thermostat chamber (WFO-450ND, EYELA) at 60°C for several days. Linear sweep voltammetry was performed from the open circuit potential to 7 V vs. Li/Li^+^. The potential range was set to 2.5 to −0.5 V vs. Li/Li^+^ for CV measurement. Linear sweep voltammetry and CV were carried out at a scan rate of 1 mV s^−1^ with an electrochemical measurement system (HZ-5000 and HZ-7000, Hokuto Denko) at 70°C in a constant climate cabinet (LU-114, ESPEC).

The ionic conductivity of each PE was measured by electrochemical impedance spectroscopy using an SP-150 potentio/galvanostat (BioLogic). The polymer electrolyte was placed between two stainless-steel electrodes separated by a PTFE spacer, and the cell temperature was controlled from 20 to 100°C with a constant climate cabinet (SU-242, ESPEC). All the measurements were performed after holding the temperature for at least 1 h. The diameter of the semicircles observed in the Nyquist plot (Supplementary Figure S8A) was defined as the electrolyte resistance (*R*_*Bulk*_). The ionic conductivity (σ) was calculated with the following equation:

$$\sigma =\frac{l}{R_{\mathrm{Bulk}}\cdot A}$$ (1)

where *l* and *A* are the thickness and the surface area of the electrolyte, respectively.

The transference numbers of lithium-ion (*t*_Li+_) in the PEs were determined using the electrochemical method proposed by Bruce and Vincent (Evans et al., 1987; Abraham et al., 1997). The required parameters were obtained using potentiostatic polarization and electrochemical impedance spectroscopy with a multi-electrochemical measurement system (HZ-Pro S4, Hokuto Denko). The polymer electrolytes infiltrated in glass filter paper (GA-55, ADVANTEC) were placed between two lithium-metal foil electrodes in a 2032-type cell. The cell was stored in a thermostat chamber (WFO-450ND, EYELA) at 60°C for several days for stabilization. Electrochemical impedance spectroscopy was performed before and after the potentiostatic polarization at 70°C in a constant climate cabinet (SU-222, ESPEC). The following equation gives the transference number of lithium-ion (*t*_Li+_):

$$t_{Li+}=\;\frac{I_{\text{SS}}R_{\text{e}}^{\text{f}}\left(\Delta V-I_{0}R_{\text{I}}^{\text{i}}\right)}{I_{0}R_{\text{e}}^{\text{i}}\left(\Delta V-I_{\text{SS}}R_{\text{I}}^{\text{f}}\right)},$$ (2)

where *ΔV* is the applied potential (Δ*V* = 10 mV), I_0_ is the initial current of the potentiostatic polarization, *I*_*SS*_ is the steady-state current of the polarization, *R*_*e*_^*i*^ is the electrolyte resistance before the polarization, and *R*_*e*_^*f*^ is the steady-state electrolyte resistance during the polarization. R_I_^i^ is the interfacial resistance before the polarization, and *R*_*I*_^*f*^ is the steady-state interfacial resistance during the polarization.

Lithium-ion transfer resistance at the lithium-metal electrode-polymer electrolyte interface was estimated by electrochemical impedance spectroscopy using a multi-electrochemical measurement system (HZ-Pro S4, Hokuto Denko). A lithium symmetric cell was used as in the *t*_Li+_ measuring 2032-type cell. The cell temperature was raised from 60 to 80°C in a constant climate cabinet (SU-222, ESPEC) and kept at the target temperature for at least 1 h before each measurement.

## Results and Discussion

### Electrochemical and Thermal Stability

**FIGURE 2:**
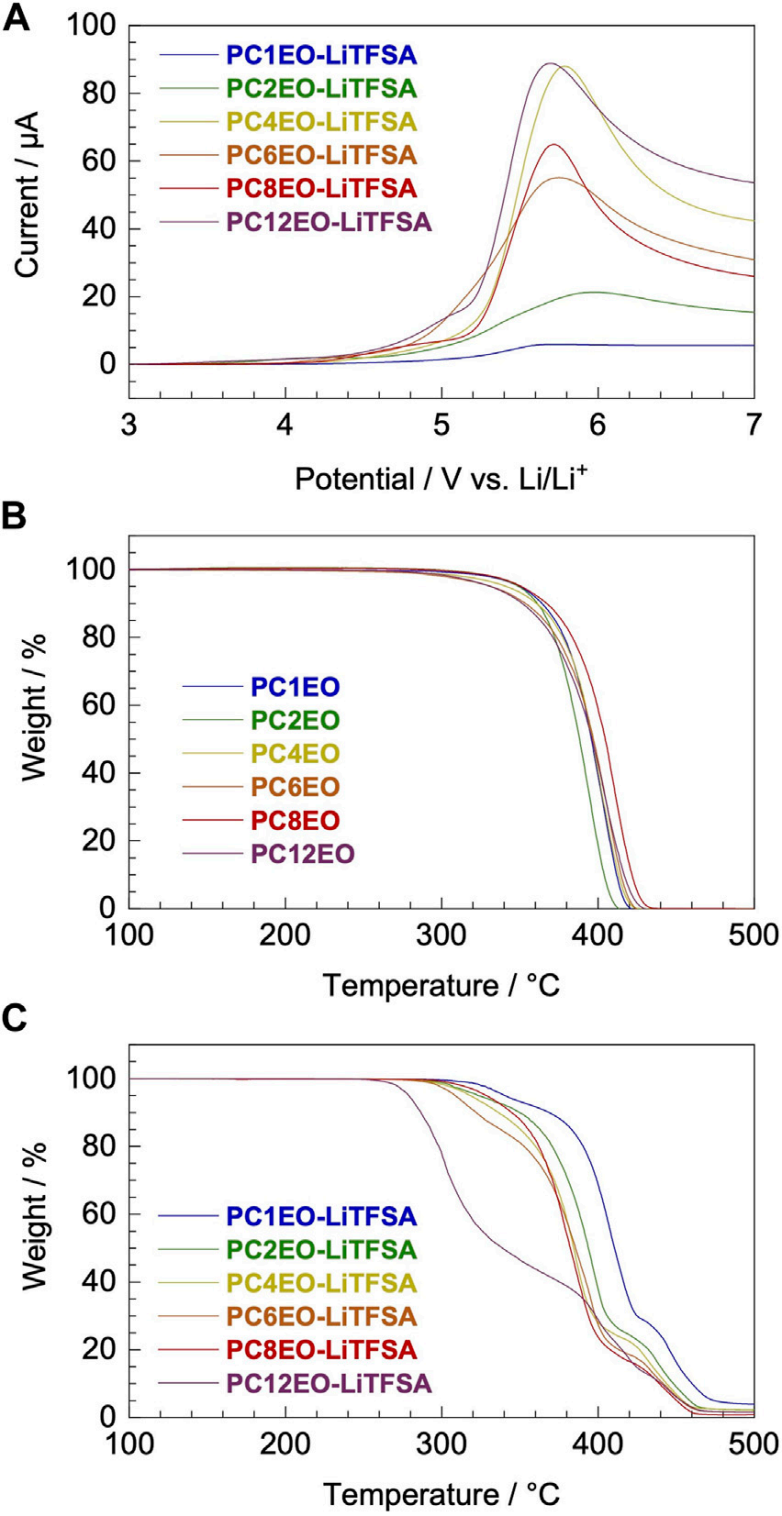
Electrochemical and thermal stability of newly synthesized PCmEO and corresponding electrolytes. **(A)** Linear sweep voltammogram from rest potential (ca. 3.0 V) to 7.0 V for PCmEO-LiTFSA on stainless steel working electrode at a scan rate of 1 mV s^−1^. Thermogravimetric (TG) curves of **(B)** PCmEO (pure polymers) and **(C)** PCmEO-LiTFSA (PEs). TG curve was obtained under He atmosphere at a heating rate of 20°C min^−1^. Thermogravimetry of the PEs was performed after pre-heating at 150°C for 10 min to remove water. The blue, green, yellow, orange, red, and purple lines show *m* = 1, 2, 4, 6, 8, and 12, respectively.

Voltammetric and thermogravimetric analysis revealed that the electrochemical and thermal stability of the newly synthesized PCmEO-LiTFSA electrolyte was partially affected by the length of the alkyl side chain. Yet, it possessed sufficient electrochemical and thermal stability for >3 V-class lithium secondary batteries which can operate up to ∼250°C (Figure 2).

The oxidation stability of PCmEO-LiTFSA was assessed with linear sweep voltammetry (LSV) at 70°C (Figure 2A, Supplementary Figure S4). Oxidation current was observed from ca. 4.0 V vs. Li/Li^+^ for PCmEO-LiTFSA (*m* = 1, 4, 6, 8, and 12), which agreed well with the oxidative decomposition potential of polyether electrolytes (Kido et al., 2015; Wang et al., 2020). Relatively low oxidation stability of ca. 3.5 V for PC2EO-LiTFSA indicates the unique inter/intramolecular interaction within the electrolyte. The cyclic voltammogram for PCmEO-LiTFSA showed stable lithium deposition/stripping reaction at ca. 0 V vs. Li/Li^+^ (Supplementary Figure S5), suggesting that PCmEO-LiTFSA electrolytes are applicable to the >3 V-class batteries (Blomgren, 2017).

Thermogravimetric analysis confirms the improvement of the thermal stability of PCmEO-LiTFSA with the shortening of the alkyl side chain (Figures 2B, C). The observed trend indicates the effect of the alkyl side chain on the interaction between dissociated ions and PCmEO, which dictates the thermal stability of the electrolytes. Thermogravimetric curves for pure PCmEO showed a plateau extending to ca. 350°C, indicating minimal weight loss and good thermal stability up to 350°C. A weight loss observed at >350°C corresponds to the evaporation of short molecules, which can be generated from the thermal decomposition of polymers initiated by the radical cleavage of the C−O or the C−C covalent bond (Costa et al., 1992). Thermal decomposition temperature (*T*_d_) of all pure PCmEO was at 370 ± 15°C, which is similar to other polyethers (e.g., *T*_d_ of PEO; ca. 400°C) (Jones et al., 1991) (Table 1). Similar *T*_d_ for all the pure PCmEO, regardless of the alkyl side-chain length, confirms the negligible effect of the alkyl side chains on the thermal decomposition process of pure PCmEO.

**TABLE 1:** List of the thermal transition temperatures obtained by thermogravimetry (TG) and differential scanning calorimetry (DSC).

m	*T*_d_/°C		*ΔT*_d_ ^a^	*T*_m_/°C		*T*_g_/°C		*ΔT*_g_ ^b^
	Pure	PEs		Pure	PEs	Pure	PEs	
1	373	383	+10	64.4	—	−34.7	−16.6	+18.1
2	368	367	−1	27.6	—	−41.9	−33.1	+8.8
4	375	359	−16	—	—	−63.5	−50.4	+13.1
6	376	352	−24	—	—	−70.3	−57.2	+13.1
8	384	356	−28	−24.7	—	−72.8	−68.3	+4.5
12	372	279	−93	−31.1	−34.8	−100.9	−95.3	+5.6

Thermal decomposition temperature (T_d_) was determined as the intersection on the baseline and diagonal line of the most significant weight loss on the TG curves. Melting temperature (T_m_) was defined as the intersection of the baseline and diagonal line of the endothermic peak on the DSC curves. Glass transition temperature (T_g_) was determined as the intersection of the baseline and the diagonal line of baseline shift on the DSC curves. The gaps of T_d_ and T_g_ between PCmEO and PCmEO-LiTFSA were described as *Δ*T_d_ and *Δ*T_g_, respectively.

a *Δ*T_d_ = T_d_ (PEs)—T_d_ (pure polymers).

b *Δ*T_g_ = T_g_ (PEs)—T_g_ (pure polymers).

The thermal decomposition temperature of PCmEO-LiTFSA (*m* ≥ 2) decreased compared to the corresponding pure PCmEO (Figures 2B, C), suggesting the acceleration of the thermal decomposition by LiTFSA. PCmEO-LiTFSA (*m* ≥ 2) was destabilized due to the interaction between ether groups and lithium-ion, weakening the C−O covalent bonds (Costa et al., 1992). Thermal decomposition temperature (*T*_d_) of PCmEO-LiTFSA (*m* ≤ 8) electrolytes showed alkyl side-chain length dependence; *m* = 1 (383°C) > *m* = 2 (367°C) > *m* = 4, 6, and 8 (359, 352, and 356°C, respectively) (Figure 2C;Table 1). The observed improvement of the thermal stability for PCmEO-LiTFSA with the shortening of the alkyl side chain (*m* ≤ 8) suggested the suppression of the radical cleavage of the covalent bond, which initiated the thermal decomposition of PCmEO-LiTFSA. The previous study on PEO electrolytes (e.g., PEO-LiCl) (Costa et al., 1992) suggests that the dissociated ion acts as a radical trap and effectively inhibits the chained radical decomposition. Therefore, the *T*_d_ trend may reflect the amount of the dissociated ion in PCmEO-LiTFSA; the amount of the dissociated ion was increased with the shortening of the alkyl side chain. On the other hand, PC12EO-LiTFSA showed an extremely low *T*_d_ of 279°C, which was inconsistent with the explanation based on the ion dissociability. We propose that the alignment of the long alkyl side chain (*m* = 12) via hydrophobic interactions hinders the approach of the dissociated ions in the vicinity of the alkyl side chain (Liu et al., 2005; Liu et al., 2006), inhibiting the dissociated ions from acting as a radical trap effectively. Further support comes from gas chromatography–mass spectrometry (GC-MS), which confirms that the decomposition of the alkyl side chain occurred before that of the polymer main chain for PC12EO-LiTFSA, unlike for PC1EO-LiTFSA (Supplementary Figure S6).

Subsequent weight loss was observed at 410−440°C (*m* ≥ 2) and >440°C (*m* = 1) for PCmEO-LiTFSA (Figure 2C), which corresponds to the thermal decomposition of the TFSA anion and the polymer residue. The assignment was supported by 1) the detection of the TFSA anion and a trace amount of polymer residue at a given temperature in GC-MS (Supplementary Figure S6) and 2) the excellent agreement with the weight loss and the added weight of TFSA in PCmEO-LiTFSA.

### Segmental Mobility

**FIGURE 3:**
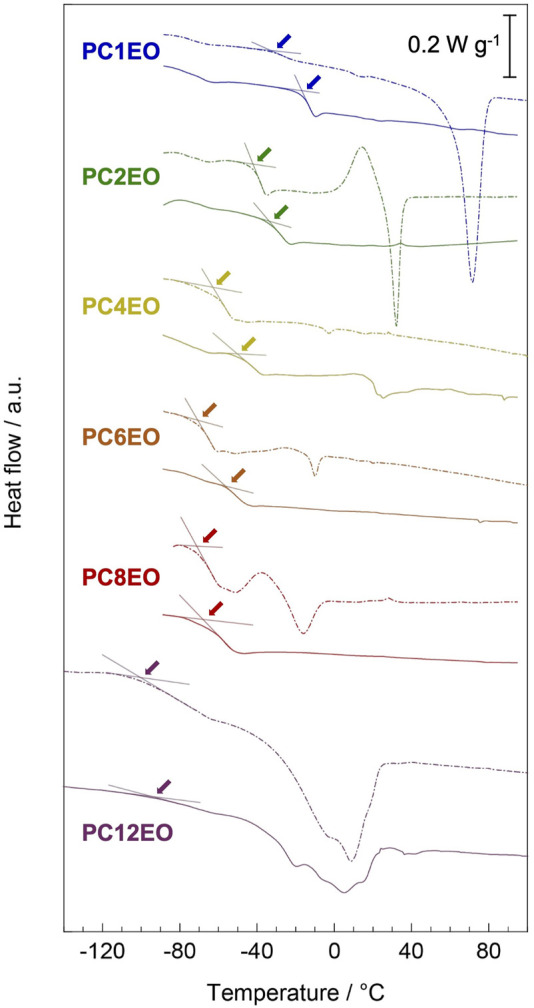
Differential scanning calorimetry (DSC) curves for PCmEO (dash line) and PCmEO-LiTFSA (solid line) were recorded during the heating step from −100 to 120°C (PC12EO and PC12EO-LiTFSA were heated from −150°C) after pre-heating up to 120°C under N_2_ flow. Guidelines and arrows indicate the glass transition temperature.

Differential scanning calorimetry (DSC) revealed that the length of alkyl side chains affects the phase transition behavior of the PCmEO and the corresponding electrolytes. The PCmEO showed crystallinity for *m* = 1, 2, 8, and 12 except for moderate length alkyls (*m* = 4 and 6), and the crystallinity was confirmed only for PC12EO-LiTFSA among PEs. The extension of the alkyl side chain improved the segmental mobility; the internal plasticizing effect and the steric hindrance of the alkyl side chain are keys to enhancing the segmental mobility of the polymer and the PE, respectively (Figure 3;Table 1).

Differential scanning calorimetry curves showed distinctive endothermic peaks for pure PCmEO (*m* = 1, 2, 8, and 12), which corresponds to the transition of the polymer from the crystalline state into the state of viscous flow (Figure 3) (Liu et al., 2006; Pérez et al., 1983). The crystal melting temperature (*T*_m_) of PC1EO and PC2EO was close to the *T*_m_ of PEO (65−70°C) (Vallée et al., 1992; Chen et al., 1995), suggesting the formation of a PEO-like crystal phase. The melting temperature of pure PCmEO (*m* = 4 and 6) was not observed, indicating the inhibition of the crystal formation due to the considerable steric hindrance of the alkyl side chain. Despite long alkyl side chains, PCmEO (*m* = 8 and 12) showed *T*_m_ of −24.7°C and −31.1°C, respectively. The observation suggests the existence of the well-aligned structure of long alkyl side chains via hydrophobic interaction (Liu et al., 2005). The melting temperature was not observed for PCmEO-LiTFSA electrolytes (*m* = 1−8) due to the sterical suppression of the crystal formation by LiTFSA (Vallée et al., 1992). The unusual observation of *T*_m_ for PC12EO-LiTFSA electrolytes suggests the existence of a unique well-aligned structure even with Li salt, consistent with the exceptionally low *T*_d_ of PC12EO-LiTFSA (Figure 2C), further suggesting the strong hydrophobic interaction between long alkyl side chains.

The glass transition temperature (*T*_g_) is tied to the polymer segmental mobility, which is an important descriptor for determining the ion transport property for PE. The glass transition temperature (*T*_g_) of pure PCmEO decreased with the extension of the alkyl side chain from −34.7°C (*m* = 1) to −100.9°C (*m* = 12) (Figure 3). The observed alkyl chain length dependence of *T*_g_ can be due to the internal plasticizing effect of alkyl side chains, originating from the large free volume within polymer chains by an alkyl side chain (Mauritz et al., 1990). The addition of LiTFSA increased the *T*_g_ of PEs for 5−18°C compared to the corresponding pure PCmEO, mainly due to the multiple interactions (coordination) between ether oxygen and lithium-ion which inhibited the bond rotation within the polymer required for the smooth segmental motion (Siqueira and Ribeiro, 2005). The gap of *T*_g_ between PCmEO and PCmEO-LiTFSA (*ΔT*_g_) correlates with the length of the alkyl side chain; *m* = 1 (18.1°C) > *m* = 2, 4, and 6 (8.8°C, 13.1°C, and 13.1°C, respectively) > *m* = 8 and 12 (4.5 and 5.6°C). The difference of *ΔT*_g_ between *m* = 1 and *m* ≥ 2 suggests the inhibition of the interaction between ether group and lithium-ion by the steric hindrance of the alkyl side chain (*m* ≥ 2), which results in a relatively large gap of *T*_g_ between pure polymer and the corresponding electrolyte. The notably small *ΔT*_g_ for PC8EO and PC12EO is probably derived from the side chain aggregation or alignment in PC8EO-LiTFSA and PC12EO-LiTFSA. We hypothesize that the hydrophobic phase (mainly consisting of side-chain aggregation or alignment) determines the segmental mobility, and only a minor contribution from the hydrophilic phase on the segmental mobility, where the interaction between the ether group and lithium-ion mainly affects. Although we could not observe the alkyl side chain aggregation for PC8EO-LiTFSA, our hypothesis is partially supported by the TG and DSC analysis, confirming the side-chain alignment for PC12EO-LiTFSA (Figures 2C, 3).

### Bulk Ion Transport Property

**FIGURE 4:**
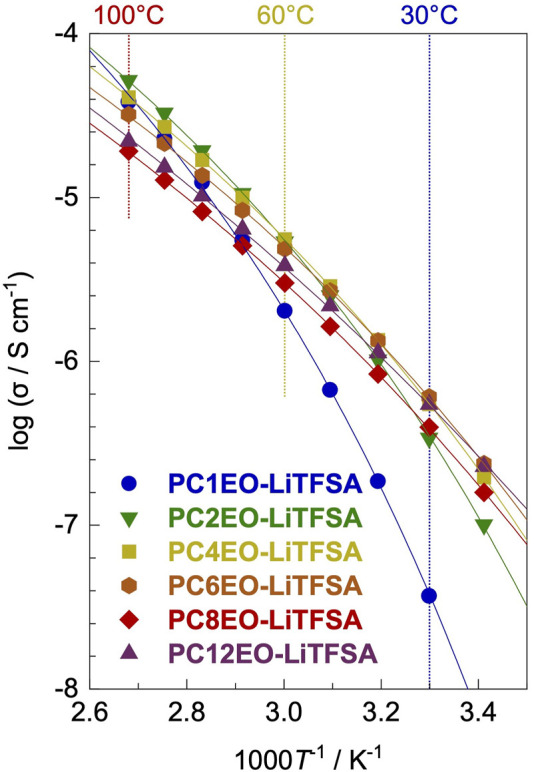
Ionic conductivities (σ) for PCmEO-LiTFSA (*m* = 1, 2, 4, 6, 8, and 12) plotted against 1000 T^−1^. Vogel–Tammann–Fulcher (VTF) fitting curves are shown in solid line. The measurements were performed at the temperature range of 20−100°C. Each symbol shows *m* = 1 (blue circle), *m* = 2 (green down triangle), *m* = 4 (yellow square), *m* = 6 (orange hexagon), *m* = 8 (red diamond), and *m* = 12 (purple triangle).

Electrochemical impedance spectroscopy (EIS) revealed that the ionic conductivity (σ) for PCmEO-LiTFSA (*m* = 1−6) at low temperature (≤30°C) increased with the extension of the alkyl side chain, while the opposite tendency was the case at high temperature (≥70°C) except for PCmEO-LiTFSA (*m* = 8 and 12) (Figure 4). The observed trend at low- and high-temperature regions can correlate with polymer segmental mobility and lithium salt dissociability, respectively. The transference number of lithium-ion was increased with a long alkyl side chain, suggesting the dominant effect of alkyl side chain length on the bulky TFSA transport. Furthermore, the aggregated/aligned alkyl side chain within PCmEO-LiTFSA (*m* = 8 and 12) was suggested to act as a rapid lithium-ion transport pathway (Table 2).

**TABLE 2:** Parameters of Vogel–Tammann–Fulcher (VTF) equation, lithium-ion transference number (*t*_Li+_), and lithium-ion conductivity (σ_Li+_) at 70°C.

m	*A*/S cm^−1^ K^1/2^	*B*/kJ mol^−1^	*T*_0_/K	*t*_Li+_	σ_Li+_ at 70°C/ × 10^−6^ S cm^−1^
1	15.03	13.6	206.6	0.15	0.84
2	3.71	12.5	190.1	0.28	2.93
4	2.92	13.7	172.8	0.30	3.02
6	1.72	13.7	166.0	0.34	2.87
8	1.72	15.3	154.9	0.37	1.90
12	7.11	19.7	127.9	0.46	2.94

*A* is a fitting parameter relevant to the number of charge carriers, *B* is the pseudo activation energy of ion conduction, and *T*_0_ is an ideal glass transition temperature, defined as *T*_0_ = T_g_ − 50. Lithium-ion transference number (t_Li+_) was obtained by electrochemical methods using chronoamperometry and electrochemical impedance spectroscopy. Lithium-ion conductivity (σ_Li+_) was calculated by multiplying transference number (t_Li+_) and ionic conductivity (σ) at 70°C.

The obtained ionic conductivity (σ) increased with the extension of the alkyl side chain (*m* = 1−6) at a low-temperature region (≤30°C) (Nyquist plots of the electrochemical impedance spectroscopy (EIS) are shown in Supplementary Figure S8A); the lowest σ for *m* = 1 (3.70 × 10^−8^ S cm^−1^) and the highest σ for *m* = 6 (6.09 × 10^−7^ S cm^−1^) at 30°C (Figure 4; Supplementary Figure S7). The observation resembles the *T*_g_ trend (Table 1), suggesting that polymer segmental motion plays a vital role in determining the ionic conductivity, especially at low temperatures (≤30°C). The Arrhenius plot of σ showed a curved shape, further supporting the hypothesis that ionic conductivity heavily depends on the polymer segmental mobility (Fan et al., 1994) similar to polyether electrolytes (Kato et al., 1990; Borodin and Smith, 2006). The ionic conductivity in the high-temperature region (≥70°C) showed an opposite trend with the alkyl side-chain length (*m* = 1−6); the lowest σ was observed for *m* = 6 (3.22 × 10^−5^ S cm^−1^) and the highest σ for *m* = 2 (5.19 × 10^−5^ S cm^−1^) at 100°C (Figure 4; Supplementary Figure S7). The result indicates that the polymer segmental mobility was not determining the ion conductivity at high temperatures, probably due to the negligible difference between the polymer segmental mobility for each polymer electrolyte (Schmidtke et al., 2015). Therefore, we hypothesize that there is a dominant factor determining the σ at high temperature (≥70°C) instead of the polymer segmental mobility.

To clarify the dominant factor determining the σ at high temperature (≥70°C), the Vogel–Tammann–Fulcher (VTF) equation (Eq. 3), i.e., a modified Arrhenius equation proposed for describing the temperature-dependence of viscosity in amorphous glasses, was employed (Angell, 2017; Sai et al., 2017b).

$$\sigma \;=\;AT^{-1/2}exp\left[-\frac{B}{R\left(T-T_{0}\right)}\right]\;.$$ (3)

Here, *A* is a parameter that relates to the number of charge carriers, *B* is the pseudo activation energy for the ion transport, *R* is the gas constant, and *T*_0_ is an ideal glass transition temperature [here, *T*_0_ = *T*_g_ − 50 K^18^ *T*_g_ from DSC measurements (Table 1)]. The experimental data showed good agreement with the fitting line for all the electrolytes (Figure 4), and the obtained VTF parameters are summarized in Table 2.

The *A* value decreased with the extension of the alkyl side chain except for PCmEO-LiTFSA (*m* = 8 and 12). We propose that the *A* value trend reflects the decrease in the salt concentration (Supplementary Table S2) and the salt dissociability. The TG analysis supported the argument that the thermal stability decreased with the extension of the alkyl side chain due to the lack of dissociated ions (Figure 2C). We concluded that the degree of salt dissociability could be a dominant factor determining the ion transport within PCmEO-LiTFSA (*m* = 1−6) at high temperatures (≥70°C).

The ionic conductivity of PCmEO-LiTFSA (*m* = 8 and 12) did not follow the above trends, showing the highest segmental mobility and the lowest salt dissociability (Figures 2C, 3). The pseudo activation energy of the ion transport (*B*) notably increased for PCmEO-LiTFSA (*m* = 8 and 12) from ca. 13 kJ mol^−1^ [PCmEO-LiTFSA (*m* = 1−6)] to >15 kJ mol^−1^. The observation suggests the unique ion transport mechanism for PCmEO-LiTFSA (*m* = 8 and 12). The ion transport mechanisms were almost identical for PCmEO-LiTFSA (*m* ≤ 6), consistent with the pseudo activation energy value of 0.125 eV (= 12.1 kJ mol^−1^) reported for PEO-LiTFSA (Das and Ghosh, 2015). Therefore, PCmEO-LiTFSA (*m* = 1−6) similarly transfers the ions to the PEO-LiTFSA system, where the ion is transferred *via* the polymer segmental motion (Borodin and Smith, 2006), including the reorganization process of a polymer chain (Matsuoka, 1997). We here propose that PCmEO-LiTFSA (*m* = 8 and 12) transfers lithium-ions through a unique ion transport pathway between aligned and/or aggregated long alkyl side chains (Liu et al., 2005). The existence of aligned and/or aggregated alkyl side chain structure was confirmed by DSC analysis (Figure 3) and is consistent with the *ΔT*_g_ trend discussed in *Segmental Mobility*. The higher ionic conductivity for PC12EO-LiTFSA than for PC8EO-LiTFSA suggests the formation of more well-aligned ion transport pathways in PC12EO-LiTFSA, consistent with the stronger hydrophobic interaction between the alkyl side chains for *m* = 12 suggested by the *T*_d_ and *T*_m_ analysis (Figures 2C, 3). Although the estimated salt dissociability of PC12EO-LiTFSA is low, the value of PC12EO-LiTFSA is relatively high, probably reflecting the higher frequency of the lithium-ion exchange from the coordination site to another through well-aligned ion transport pathways. PC8EO-LiTFSA forms incomplete ion transport pathways due to relatively weak hydrophobic interaction of the alkyl side chain compared to *m* = 12, resulting in a lower *A* value than PC12EO-LiTFSA.

Lithium transference number (*t*_Li+_) calculated by the potentiostatic polarization (Bruce-Vincent) method (details of *t*_Li+_ derivations are shown in Supplementary Figure S9; Supplementary Table S4) revealed that *t*_Li+_ increased with the extension of alkyl side chain from *m* = 1 (*t*_Li+_ = 0.15) to *m* = 12 (*t*_Li+_ = 0.46) (Table 2). The significantly low *t*_Li+_ for PC1EO-LiTFSA was comparable to that of a PEO-based electrolyte (e.g., PEO-LiTFSA, *t*_Li+_ ≤ 0.2) (Hayamizu et al., 2002; Devaux et al., 2012; Pożyczka et al., 2017), suggesting a PEO-like lithium-ion transport mechanism in PC1EO-LiTFSA. In contrast, the increase of *t*_Li+_ for PCmEO-LiTFSA with the extension of the alkyl side chain suggested the contribution of the alkyl side chain to suppress TFSA transport. The steric hindrance of the alkyl side chain selectively suppressed the transport of TFSA anion with a large ionic radius compared to lithium-ion. The highest lithium-ion conductivity (σ_Li+_) was achieved for PC4EO electrolytes among the electrolytes tested. The σ_Li+_ trend indicates that the alkyl side-chain length of *m* = 4 has the optimal balance between polymer segmental mobility, salt dissociability, and *t*_Li+_. Comparable σ_Li+_ was observed for PC12EO-LiTFSA, probably due to the formation of rapid lithium-ion transport pathways within the hydrophobic phase of the aligned alkyl side chain.

### Lithium-Ion Transfer at the Lithium-Metal Electrode-Polymer Electrolyte Interface

**FIGURE 5:**
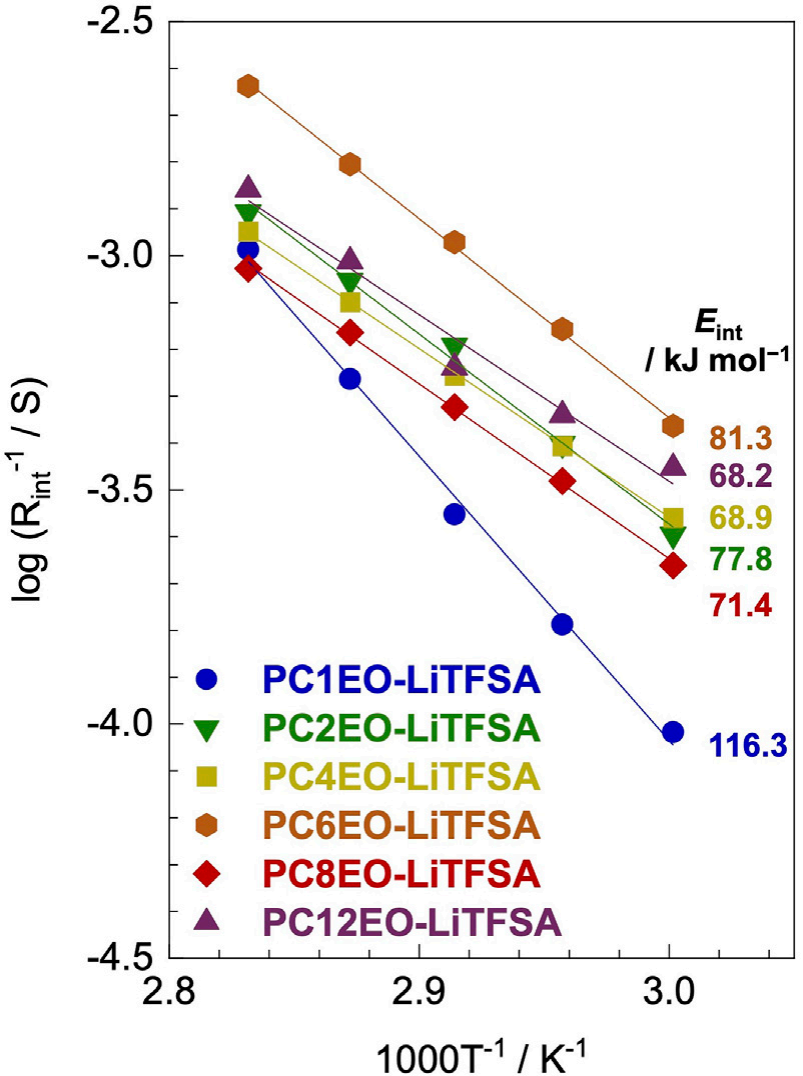
Reciprocal of lithium-metal electrode-polymer electrolyte interface resistance (*R*_int_) for PCmEO-LiTFSA electrolytes (*m* = 1, 2, 4, 6, 8, and 12) plotted against 1000 T^−1^. Arrhenius fitting lines are shown in solid lines. The measurements were performed at the temperature range of 60−80°C using a lithium symmetric cell.

The lithium-ion deposition/reduction process at the electrode-electrolyte interface also plays a significant role in the performance of the next-generation lithium secondary batteries. The activation energy of the lithium-ion transfer at the electrode-electrolyte interface (*E*_int_) partially depends on the alkyl side-chain length; PC1EO-LiTFSA showed a notably high *E*_int_ of 116.3 kJ mol^−1^ while the rest of PCmEO-LiTFSA (*m* ≥ 2) showed an *E*_int_ of 68−82 kJ mol^−1^ (Figure 5).

The Arrhenius plot of the interface resistance (*R*_int_) showed a linear relationship, suggesting that the polymer segmental motion was not involved in the lithium-ion transfer at the electrode-electrolyte interface (Figure 5). The activation energy of lithium-ion transfer at the electrode-electrolyte interface (*E*_int_) (Abe et al., 2004; Abe et al., 2005; Minato and Abe, 2017) became higher in PCmEO-LiTFSA (≥68 kJ mol^−1^) than in liquid electrolytes (53−59 kJ mol^−1^), where the process is dominated by the (de-)solvation process of the lithium-ion (Abe et al., 2004). The trend in *E*_int_ suggested the sluggish (de-)coordination process within the polymer matrix compared to that in liquid electrolytes. PC1EO-LiTFSA showed the highest *E*_int_ of 116.3 kJ mol^−1^ among the PCmEO electrolytes, while the PCmEO-LiTFSA (*m* ≥ 2) showed *E*_int_ within the range of 68−82 kJ mol^−1^. We here propose that the *E*_int_ reflects the stability of the lithium-ion coordination structure, closely related to the strength of the interaction between ether oxygen and lithium-ion. The notably high *E*_int_ value of PC1EO-LiTFSA thus indicates the stable structure around lithium-ion, which was consistent with the largest *ΔT*_g_ for PC1EO, suggesting the multiple and stable interaction between the lithium-ion and ether group (Table 1).

### Interactions Around Lithium-Ion

**FIGURE 6:**
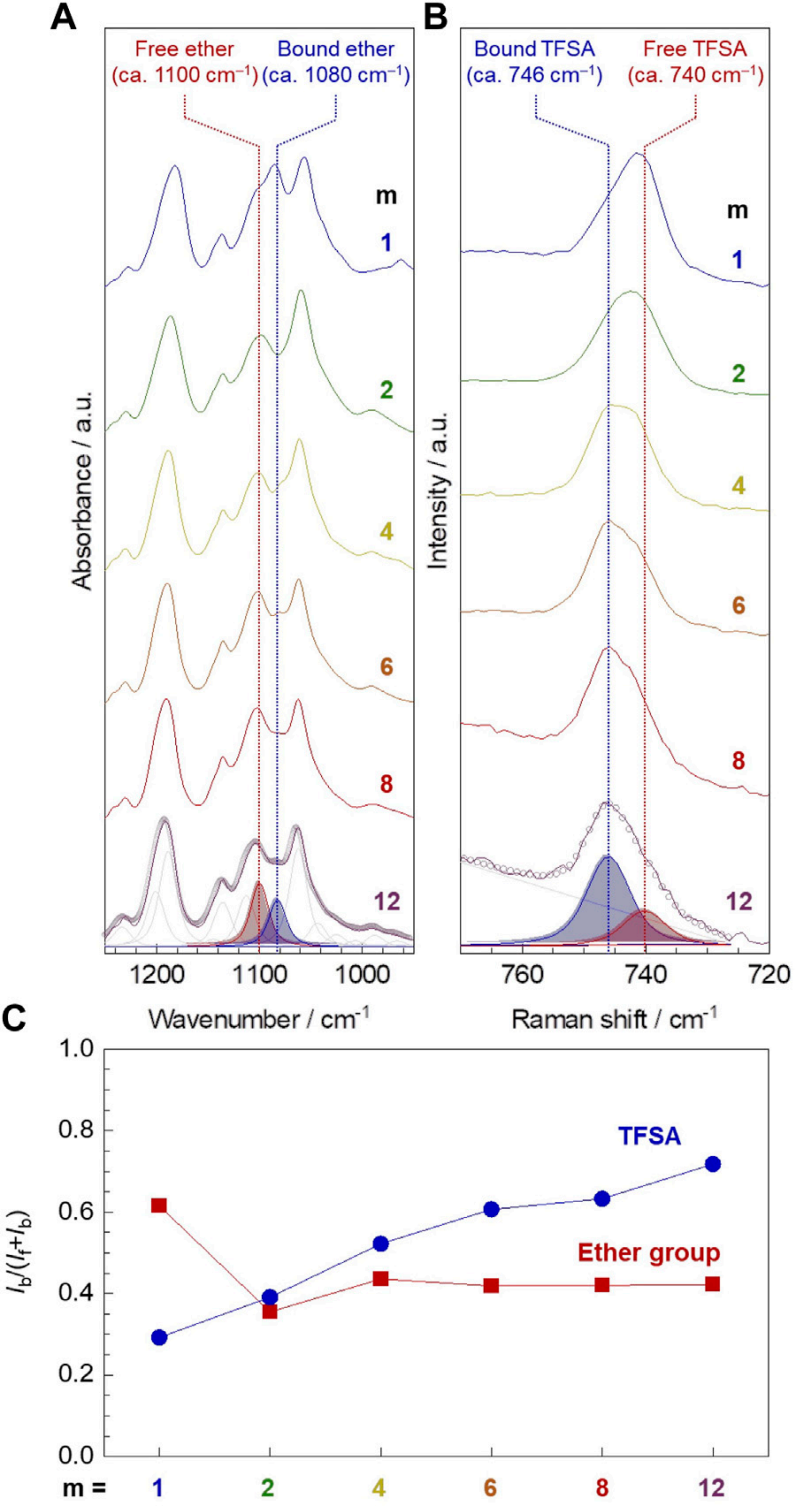
Interaction between Li^+^ and ether group/TFSA anion in PCmEO-LiTFSA. **(A)** Infrared (IR) spectra of ν_as_ (COC) region and **(B)** Raman spectra of δ_s_ (CF_3_) region (solid line). The alkyl side chain becomes longer in order from top to bottom; *m* = 1, 2, 4, 6, 8, and 12. Peak deconvolution was performed by PeakFit software using pseudo-Voigt functions with a fixed half-width at half-maximum, and representative deconvoluted peaks are shown for PC12EO-LiTFSA. The open circle shows typical curve-fitting results. **(C)** Alkyl side chain length dependency of the ratio of the peak area assigned to polar groups (red square for ether groups and blue circle for TFSA anion) interacting with lithium-ion (*I*_b_) to the total peak area (*I*_f_ + *I*_b_).

The infrared (IR) and Raman spectroscopic analysis confirmed that the amount of ether oxygen interacting with lithium-ion was notably larger for PC1EO-LiTFSA compared to PCmEO-LiTFSA (*m* ≥ 2), whereas the number of TFSA anion interacting with lithium-ion was increased with the extension of the alkyl side chain (Figure 6).

Infrared (IR) spectra for pure PCmEO showed an intense peak at 1094−1100 cm^−1^, which can be assigned to ν_as_ (COC) of ether groups on the main and/or side chain (Supplementary Figures S11, S12) (Matsuura and Fukuhara, 1986). The shouldered peak was observed in the ν_as_ (COC) region for the corresponding PCmEO-LiTFSA (Figure 6A; Supplementary Figure S13), which could be due to the overlapping of the peaks assigned to the ether group without any interactions (denoted as free ether) (ca. 1100 cm^−1^) and the ether group interacting with lithium-ion (denoted as bound ether) (ca. 1080 cm^−1^). A clear peak shift from 1095 to 1084 cm^−1^ was confirmed by introducing excessive lithium iodide in PC1EO (Supplementary Figure S14), which validates that the newly appeared peak (1084 cm^−1^) corresponds to the ν_as_ (COC) of bound ether. Further support came from the redshift of the peak at 1066 cm^−1^ of ν_as_ (COC) of pure tetrahydrofuran to 1043 cm^−1^ for neat LiTFSA solution in tetrahydrofuran (Supplementary Figure S14), indicating the weakening of the C−O bond due to the interaction between ether oxygen and lithium-ion (Wieczorek et al., 1998; Gámez et al., 2011). The ratio of bound ether to all ether groups, i.e., bound and free ether groups, was calculated by comparing the deconvoluted ν_as_ (COC) peak area. The ratio of bound ether became the largest for *m* = 1 compared to *m* = 2−12 (Figure 6C, red square symbol), suggesting the effective inhibition of the interaction between ether oxygen and lithium-ion by the sterically hindered alkyl side chain (*m* ≥ 2) (Sai et al., 2017b). However, the ratio of the bound ether remained almost the same for PCmEO-LiTFSA with *m* ≥ 2, probably due to the hydrophobic exclusion, which inhibits the approach of the alkyl side chain to the vicinity of the hydrophilic Li^+^-coordination site. As a result, the ethyl(-ene) side chain (*m* = 2) connected to ether groups could stay in the vicinity of the ether group, which effectively inhibits the interaction between lithium-ion and ether oxygen.

Raman spectra showed an intense peak at 740−746 cm^−1^, corresponding to δ_s_ (CF_3_) of the TFSA anion (Figure 6B) (Rey et al., 1998). The δ_s_ (CF_3_) peak can be deconvoluted into two peaks, which were assigned to TFSA anion with (bound TFSA, 746 cm^−1^) and without (free TFSA, 740 cm^−1^) interacting with lithium-ion (Umebayashi et al., 2007; Sai et al., 2017a). The increase in the amount of bound TFSA suggests the decreased lithium salt dissociability for PCmEO-LiTFSA in line with the extension of the alkyl side chain, which confirms the salt dissociability trend proposed by TG and VTF (*A* value) analysis. The decrease in salt dissociability was probably due to the reduction of the dielectric constant by increasing the non-polarity with the extension of the alkyl side chain (Wheatle et al., 2017).

**FIGURE 7:**
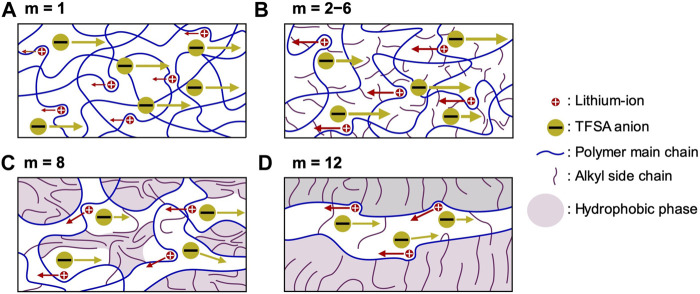
Proposed model for ion transportation within PCmEO-LiTFSA. **(A)** *m* = 1; ether group interacts well with lithium-ion and isolates the TFSA anion, **(B)** *m* = 2−6; alkyl side chain dispersed and suppressed the interaction between ether group and lithium-ion as well as TFSA anion and lithium-ion, **(C)** *m* = 8; alkyl side chain partially aggregates and inhibits the transport of ions, and **(D)** *m* = 12; alkyl side chain aligned via hydrophobic interaction and provide rapid lithium-ion transport pathways. Red and yellow circles correspond to the lithium-ion and TFSA anion, respectively. Blue and purple curves represent polymer main chain with ether group and alkyl side chain, respectively. The purple area shows a hydrophobic phase with alkyl side chain aggregation.

The electrolyte properties (including thermal stability, thermal phase transition, segmental mobility, salt dissociability, ionic conductivity, lithium-ion transference number, and lithium-ion transfer at the electrode-electrolyte interface) are heavily affected by the length of the alkyl side chain (Figure 7).

The short alkyl side chain (*m* = 1) with minor steric hindrance showed a negligible effect on the interaction between ether groups and lithium-ion, forming a coordination structure similar to that of PEO electrolytes. The stable coordination structure of PC1EO-LiTFSA significantly increases the glass transition temperature (Figure 3; Table 1) and the activation energy of lithium-ion transfer at the electrode-electrolyte interface (*E*_int_) (Figure 5). On the other hand, the relatively large steric hindrance of the alkyl side chain (*m* = 2−6) effectively suppressed the interaction between ether oxygen and lithium-ion, which corresponds to the smaller *ΔT*_g_ and *E*_int_ than PC1EO-LiTFSA (Table 1; Figure 5). Although a long alkyl side chain (*m* = 8 and 12) suppressed the interaction between ether oxygen and lithium-ion, it can be partially aligned and/or aggregated due to the large hydrophobic interaction between the side chains, decreasing *ΔT*_g_ compared to PCmEO-LiTFSA (*m* ≤ 6). In addition, the alkyl side chains with *m* = 12 aligned well in the electrolyte, creating rapid lithium-ion transport pathways and giving relatively high lithium-ion conductivity (σ_Li+_) (Table 2).

The unique character of the alkyl side chain associated with the extension of the alkyl groups also plays a significant role in determining the electrolyte properties. Polymer segmental mobility increased with the extension of the alkyl side chain (Table 1; Figure 3) due to the increase in the internal plasticizing effect of the alkyl side chain. The extension of the alkyl side chain also decreased the LiTFSA dissociability by reducing the overall dielectric constant of electrolytes derived from the increased contribution from the non-polar alkyl side chain. The decrease in the number of dissociated ions with the extension of the alkyl side chain decreases the thermal stability (Table 1) and the ionic conductivity at high temperatures (>70°C) (Table 2). The transference number of lithium-ion (*t*_Li+_) increased with the extension of the alkyl side chain due to the effective inhibition of the transport of bulky TFSA anion by significant steric hindrance of the alkyl side chain. The balance between polymer segmental mobility, salt dissociability, and the transference number of lithium-ion decides the σ_Li+_; alkyl side chain with moderate chain length (*m* = 4) showed the highest σ_Li+_ of 3.02 × 10^−6^ S cm^−1^ at 70°C among the electrolytes tested in this study.

## Conclusion

The influence of alkyl side-chain length on the interaction between lithium-ion and ether groups, as well as the lithium-ion transport within polyether-based electrolytes, was clarified by thermal, electrochemical, and spectroscopic analysis. The length of the alkyl side chain significantly affects the lithium coordination structure; the electrolyte with a short alkyl side chain (*m* = 1) has the most stable lithium coordination structure surrounded by ether oxygen, while the extension of the alkyl side chain (*m* = 2−12) effectively suppressed the interaction between ether oxygen and lithium-ion, leading to a decrease in the glass transition temperature and the activation energy of lithium-ion transfer at the electrode-electrolyte interface. Various characteristics of the alkyl side chain associated with the extension of the alkyl groups also play a significant role in determining the electrolyte properties. The increase in the internal plasticizing effect of the alkyl side chain increases the polymer segmental mobility. The decrease in the overall dielectric constant of electrolytes derived from the increased contribution from the non-polar alkyl side chain decreases the LiTFSA dissociability. The decrease in the number of dissociated ions with the extension of the alkyl side chain decreases the thermal stability and the ionic conductivity at high temperatures (>70°C). The transference number of lithium-ion (*t*_Li+_) increased with the extension of the alkyl side chain due to the effective inhibition of the transport of bulky TFSA anion by significant steric hindrance of the alkyl side chain. Furthermore, the hydrophobic phase with aligned and/or aggregated long alkyl side chains (*m* = 12) acts as a rapid lithium-ion transport pathway, leading to high lithium-ion conductivity of 2.94 × 10^−6^ S cm^−1^. Alkyl side chain with medium length (*m* = 4) showed the highest lithium-ion conductivity of 3.02 × 10^−6^ S cm^−1^ due to the optimized balance between polymer segmental mobility, salt dissociability, and the transference number of lithium-ion. The result thus emphasizes the importance of optimizing the length of the alkyl side chain to improve (electro)chemical properties of PE. The proposed polymer design strategy can be combined with existing strategies, including but not limited to the composition tuning of copolymer matrices and the topology design of non-linear polymers, further increasing the lithium conductivity to meet the requirements for implementing the all-solid rechargeable battery technologies.

## Data Availability

The original contributions presented in the study are included in the article/Supplementary Material; further inquiries can be directed to the corresponding author.
