# Supplementary material for: Effect of Alkyl Side Chain Length on the Lithium-Ion Conductivity for Polyether Electrolytes
Source: Front Chem. 2022 Jul 14;10:943224. doi: 10.3389/fchem.2022.943224 (PMC9329624; doi:10.3389/fchem.2022.943224)
Supplement: Supplementary file 1 [file DataSheet1.pdf]

Supplementary Material

Preparation of monomer

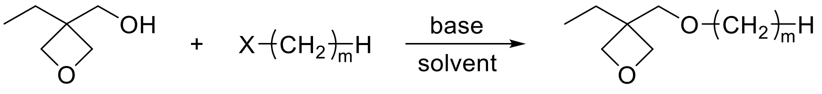

Figure S1 Synthesis of 3-ethyl-3-(*alkyl*)oxymethyloxetane (CmEO, m = 1, 2, 4, 6, 8, and 12). X stands for the following leaving groups; iodo, bromo, or methanesulfonyl group.

Each oxetane derivative was synthesized from 3-ethyl-3-hydroxymethyloxetane (EHO) by Williamson-ether reaction.

(**m = 1**) 3-ethyl-3-methyloxymethyloxetane (C1EO) was synthesized from EHO (8.75 mL, 0.075 mol), iodomethane (6.23 mL, 0.10 mol), and 40−60 wt% sodium hydride (4.11 g, 0.10 mol). Sodium hydride dispersed in mineral oil was washed with hexane prior to use. Sodium hydride and EHO were dissolved in 150 mL of tetrahydrofuran (THF) at room temperature under an argon atmosphere, subsequently heated to 50 °C to activate EHO. Iodomethane was then added to the activated EHO solution and refluxed overnight for Williamson-ether reaction. THF in the obtained mixture was removed using a rotary evaporator, subsequently diluted with dichloromethane, and washed with water. The obtained organic extract was dehydrated with magnesium sulfate, followed by the removal of dichloromethane by distillation. The residue was distilled under reduced pressure (150 °C, 150−200 hPa) by Kugelrohr. Yield: 6.17 g, 63.2%.

C1EO ^{1}H NMR (δ, ppm from tetramethylsilane (TMS) in $CDCl_3$): 0.89 (t, 3H, J = 7.3 Hz, $-CH_2-C\underline{H}_3$), 1.74 (q, 2H, J = 7.3 Hz, $-C\underline{H}_2-CH_3$), 3.40 (s, 3H, $-O-C\underline{H}_3$), 3.51 (s, 2H, $-C\underline{H}_2-O-CH_3$), 4.42 (dd, 4H, J = 32.0 Hz, 5.8 Hz, ring, $-C\underline{H}_2-O-C\underline{H}_2-$).

C1EO ^{13}C NMR (δ, ppm from $CDCl_3$ (77.0 ppm)): 7.99 ($-CH_2-\underline{C}H_3$), 26.54 ($-\underline{C}H_2-CH_3$), 43.15 ($>\underline{C}<$), 59.14 ($-O-\underline{C}H_3$), 73.30 ($-\underline{C}H_2-O-CH_3$), 78.27 ($-\underline{C}H_2-O-\underline{C}H_2-$).

(**m = 2**) 3-ethyl-3-ethyloxymethyloxetane (C2EO) was synthesized from EHO (8.75 mL, 0.075 mol), bromoethane (7.46 mL, 0.10 mol) and 40−60 wt% sodium hydride (4.00 g, 0.10 mol). Sodium hydride dispersed in mineral oil was washed with hexane prior to use. Sodium hydride and EHO were dissolved in 150 mL of THF at room temperature under argon atmosphere, subsequently heated to 50 °C to activate EHO. Bromoethane was then added to the activated EHO solution and refluxed overnight for Williamson-ether reaction. THF in the obtained mixture was removed using a rotary evaporator, subsequently diluted with dichloromethane, and washed with water. The obtained organic extract was dehydrated with magnesium sulfate, followed by the removal of dichloromethane. The distillate residue was applied to a silica column (eluent = ethyl acetate: hexane, 5: 5 (vol)). After removing the eluent by distillation, the residue was distilled under reduced pressure (150 °C, 280−240 hPa) by Kugelrohr. Yield: 3.70 g, 34.2%.

C2EO ^{1}H NMR (δ, ppm from TMS in $CDCl_3$): 0.89 (t, 3H, J = 7.3 Hz, $\geq C-CH_2-C\underline{H}_3$), 1.21 (t, 3H, J = 7.0 Hz, $-O-CH_2-C\underline{H}_3$), 1.75 (q, 2H, J = 7.3 Hz, $\geq C-C\underline{H}_2-CH_3$), 3.53 (q, 2H, J = 7.2 Hz, −O

$-C\underline{H}_2-CH_3$), 3.54 (s, 2H, $-C\underline{H}_2-O-CH_2-CH_3$) 4.43 (dd, 4H, *J* = 31.3 Hz, 5.8 Hz, ring, $\geq C-C\underline{H}_2-O-C\underline{H}_2-C\leq$).

C2EO ^{13}C NMR (δ, ppm from $CDCl_3$ (77.0 ppm)): 8.08 ($\geq C-CH_2-\underline{C}H_3$), 14.94 ($-O-CH_2-\underline{C}H_3$), 26.60 ($\geq C-\underline{C}H_2-CH_3$), 43.23 ($>\underline{C}<$), 66.72 ($-O-\underline{C}H_2-CH_3$), 73.02 ($-\underline{C}H_2-O-CH_2-CH_3$), 78.51 ($\geq C-\underline{C}H_2-O-\underline{C}H_2-C\leq$).

(**m = 4**) Butyl methanesulfonate (BuOMs) was synthesized from butanol (22.9 mL, 0.25 mol) and methanesulfonyl chloride (19.3 mL, 0.25 mol). Butanol and methanesulfonyl chloride were dissolved in co-solvent consist of 104 mL of triethylamine and 200 mL of dichloromethane, subsequently stirred for 5 h at 0 °C for the butanol mesylation. The reaction mixture was washed with water and aqueous 1M HCl. The obtained organic extract was dehydrated with magnesium sulfate, followed by the removal of dichloromethane by distillation. Yield: 31.86 g, 83.7 %.

BuOMs ^{1}H NMR (δ, ppm from TMS in $CDCl_3$): 0.95 (t, 3H, *J* = 7.3 Hz, $-CH_2-C\underline{H}_3$), 1.44 (sext., 2H, *J* = 7.3 Hz, $-C\underline{H}_2-CH_3$), 1.73 (quin., 2H, *J* = 7.3 Hz, $-C\underline{H}_2-CH_2-CH_3$), 3.02 (s, 3H, $-SO_2-C\underline{H}_3$), 4.24 (t, 2H, *J* = 6.7 Hz,$-O-C\underline{H}_2-$).

3-ethyl-3-butyloxymethyloxetane (C4EO) was synthesized from EHO (10.10 mL, 0.087 mol), BuOMs (9.93 mL, 0.072 mol), and 40−60 wt% sodium hydride (4.12 g, 0.10 mol). Sodium hydride dispersed in mineral oil was washed with hexane prior to use. Sodium hydride, EHO, and BuOMs were dissolved in 150 mL of THF at room temperature under argon atmosphere, subsequently heated to 50 °C, and refluxed overnight for Williamson-ether reaction. THF in the obtained mixture was removed using a rotary evaporator, subsequently diluted with dichloromethane, and washed with water. The obtained organic extract was dehydrated with magnesium sulfate, followed by the removal of dichloromethane by distillation. The residue was distilled under reduced pressure (150 °C, 200−150 hPa) by Kugelrohr. Yield: 9.40 g, 75.6%.

C4EO ^{1}H NMR (δ, ppm from TMS in $CDCl_3$): 0.92 (m, 6H, $\geq C-CH_2-C\underline{H}_3$, $-CH_2-CH_2-C\underline{H}_3$), 1.37 (sext., 2H, *J* = 6.7 Hz, $-CH_2-C\underline{H}_2-CH_3$), 1.56 (quin., 2H, *J* = 6.7 Hz, $-CH_2-C\underline{H}_2-CH_2-$), 1.74 (q, 2H, *J* = 7.3 Hz, $\geq C-C\underline{H}_2-CH_3$), 3.46 (t, 2H, *J* = 6.7 Hz, $-O-C\underline{H}_2-CH_2-$), 3.53 (s, 2H, $-C\underline{H}_2-O-CH_2-CH_2-$), 4.39 (dd, 4H, *J* = 34.8 Hz, 6.1 Hz, ring, $\geq C-C\underline{H}_2-O-C\underline{H}_2-C\leq$).

C4EO ^{13}C NMR (δ, ppm from $CDCl_3$ (77.0 ppm)): 8.19 ($\geq C-CH_2-\underline{C}H_3$), 13.90 ($-CH_2-CH_2-\underline{C}H_3$), 19.32 ($-CH_2-\underline{C}H_2-CH_3$), 26.71 ($\geq C-\underline{C}H_2-CH_3$), 31.64 ($-CH_2-\underline{C}H_2-CH_2-$), 43.40 ($>\underline{C}<$), 71.30 ($-O-\underline{C}H_2-CH_2-$), 73.34 ($-\underline{C}H_2-O-CH_2-CH_2-$), 78.63 ($\geq C-\underline{C}H_2-O-\underline{C}H_2-C\leq$).

(**m = 6**) 3-ethyl-3-hexyloxymethyloxetane (C6EO) was synthesized from EHO (5.70 mL, 0.050 mol), 1-bromohexane (6.96 mL, 0.050 mol), and tetrabutylammonium bromide (TBAB) (1.62 g, 0.0050 mol). EHO, 1-bromohexane and TBAB were dissolved in co-solvent consist of 250 mL of toluene and 250 mL of aqueous 40wt% NaOH. The solution refluxed overnight at 60 °C with a strong stir for Williamson-ether reaction. The obtained reaction mixture washed with water, subsequently dehydrated with magnesium sulfate, and then toluene was removed by distillation. The residue was distilled under reduced pressure (100 °C, 1.3 hPa) by Kugelrohr. Yield: 5.90 g, 58.9%.

C6EO ^{1}H NMR (δ, ppm from TMS in $CDCl_3$): 0.85−0.93 (m, 6H, $\geq C-CH_2-C\underline{H}_3$, $-CH_2-CH_2-C\underline{H}_3$), 1.24−1.38 (m, 6H, $-C\underline{H}_2-C\underline{H}_2-C\underline{H}_2-CH_3$), 1.57 (quin., 2H, *J* = 6.7 Hz, $-O-CH_2-C\underline{H}_2-$), 1.74 (q, 2H, *J* = 7.3 Hz, $\geq C-C\underline{H}_2-CH_3$), 3.45 (t, 2H, *J* = 6.7 Hz, $-O-C\underline{H}_2-CH_2-$), 3.52 (s, 2H, $-C\underline{H}_2-O-CH_2-CH_2-$) 4.39 (dd, 4H, *J* = 34.3 Hz, 6.1 Hz, ring, $\geq C-C\underline{H}_2-O-C\underline{H}_2-C\leq$).

C6EO ^{13}C NMR (δ, ppm from $CDCl_3$ (77.0 ppm)): 8.17 ($\geq C-CH_2-\underline{C}H_3$), 14.09 ($-CH_2-CH_2-\underline{C}H_3$), 22.59 ($-CH_2-\underline{C}H_2-CH_3$), 26.70 ($\geq C-\underline{C}H_2-CH_3$), 29.19 ($-\underline{C}H_2-CH_2-CH_2-CH_3$), 29.46 ($-\underline{C}H_2-CH_2-CH_3$), 31.62 ($-O-CH_2-\underline{C}H_2-$), 43.37 ($>\underline{C}<$), 71.61 ($-O-\underline{C}H_2-CH_2-$), 73.32 ($-\underline{C}H_2-O-CH_2-CH_2-$), 78.62 ($\geq C-\underline{C}H_2-O-\underline{C}H_2-C\leq$).

(**m = 8**) 3-ethyl-3-octyloxymethyloxetane (C8EO) was synthesized from EHO (6.84 mL, 0.070 mol), 1-bromooctane (8.64 mL, 0.050 mol), and TBAB (1.62 g, 0.0050 mol). EHO, 1-bromooctane, and TBAB were dissolved in co-solvent consist of 200 mL of toluene and 200 mL of aqueous 40wt% NaOH. The solution refluxed for 3 days at 70 °C with a strong stir for Williamson-ether reaction. The obtained reaction mixture washed with water, subsequently dehydrated with magnesium sulfate, and then toluene was removed by distillation. The residue was distilled under reduced pressure (150 °C, 5.3 hPa) by Kugelrohr. Yield: 4.02 g, 35.2%.

C8EO ^{1}H NMR (δ, ppm from TMS in $CDCl_3$): 0.85−0.93 (m, 6H, $\geq C-CH_2-C\underline{H}_3$, $-CH_2-CH_2-C\underline{H}_3$), 1.22−1.37 (m, 10H, $-C\underline{H}_2-C\underline{H}_2-C\underline{H}_2-C\underline{H}_2-C\underline{H}_2-CH_3$), 1.57 (quin., 2H, J = 6.7 Hz, $-O-CH_2-C\underline{H}_2-$), 1.74 (q, 2H, J = 7.3 Hz, $\geq C-C\underline{H}_2-CH_3$), 3.44 (t, 2H, J = 6.7 Hz, $-O-C\underline{H}_2-CH_2-$), 3.52 (s, 2H, $-C\underline{H}_2-O-CH_2-CH_2-$) 4.38 (dd, 4H, J = 34.3 Hz, 6.1 Hz, ring, $\geq C-C\underline{H}_2-O-C\underline{H}_2-C\leq$).

C8EO ^{13}C NMR (δ, ppm from $CDCl_3$ (77.0 ppm)): 8.19 ($\geq C-CH_2-\underline{C}H_3$), 14.06 ($-CH_2-CH_2-\underline{C}H_3$), 22.62 ($-CH_2-\underline{C}H_2-CH_3$), 26.10 ($-O-CH_2-CH_2-\underline{C}H_2-$), 26.70 ($\geq C-\underline{C}H_2-CH_3$), 29.25 ($-O-CH_2-CH_2-CH_2-\underline{C}H_2-$), 29.38 ($-\underline{C}H_2-CH_2-CH_2-CH_3$), 29.50 ($-\underline{C}H_2-CH_2-CH_3$), 31.80 ($-O-CH_2-\underline{C}H_2-$), 43.37 ($>\underline{C}<$), 71.61 ($-O-\underline{C}H_2-CH_2-$), 73.32 ($-\underline{C}H_2-O-CH_2-CH_2-$), 78.61 ($\geq C-\underline{C}H_2-O-\underline{C}H_2-C\leq$).

(**m = 12**) 3-ethyl-3-dodecyloxymethyloxetane (C12EO) was synthesized from EHO (6.84 mL, 0.070 mol), 1-Iododecane (10.74 mL, 0.050 mol), and 40−60 wt% sodium hydride (3.37 g, 0.070 mol). Sodium hydride dispersed in mineral oil was washed with hexane prior to use. Sodium hydride and EHO were dissolved in 400 mL of THF at room temperature under argon atmosphere, subsequently heated to 60 °C to activate EHO. 1-Iododecane was then added to the activated EHO solution and refluxed for 1 day for Williamson-ether reaction. THF in the obtained mixture was removed using a rotary evaporator, subsequently diluted with chloroform, and washed with water. The obtained organic extract was dehydrated with magnesium sulfate, followed by the removal of chloroform by distillation. The distilled residue was applied to a silica column (eluent = ethyl acetate: hexane, 1: 9 (vol)), and then the eluent was removed by distillation. Yield: 5.60 g, 39.4%.

C12EO ^{1}H NMR (δ, ppm from TMS in $CDCl_3$): 0.86−0.91 (m, 6H, $\geq C-CH_2-C\underline{H}_3$, $-CH_2-CH_2-C\underline{H}_3$), 1.21−1.36 (m, 18H, $-C\underline{H}_2-C\underline{H}_2-C\underline{H}_2-C\underline{H}_2-C\underline{H}_2-C\underline{H}_2-C\underline{H}_2-C\underline{H}_2-C\underline{H}_2-CH_3$), 1.57 (quin., 2H, J = 6.7 Hz, $-O-CH_2-C\underline{H}_2-$), 1.74 (q, 2H, J = 7.3 Hz, $\geq C-C\underline{H}_2-CH_3$), 3.45 (t, 2H, J = 6.7 Hz, $-O-C\underline{H}_2-CH_2-$), 3.52 (s, 2H, $-C\underline{H}_2-O-CH_2-CH_2-$) 4.39 (dd, 4H, J = 34.3 Hz, 6.1 Hz, ring, $\geq C-C\underline{H}_2-O-C\underline{H}_2-C\leq$).

C12EO ^{13}C NMR (δ, ppm from $CDCl_3$ (77.0 ppm)): 8.16 ($\geq C-CH_2-\underline{C}H_3$), 14.09 ($-CH_2-CH_2-\underline{C}H_3$), 22.66 ($-CH_2-\underline{C}H_2-CH_3$), 26.10 ($-O-CH_2-CH_2-\underline{C}H_2-$), 26.70 ($\geq C-\underline{C}H_2-CH_3$), 29.51 ($-\underline{C}H_2-\underline{C}H_2-\underline{C}H_2-\underline{C}H_2-\underline{C}H_2-\underline{C}H_2-\underline{C}H_2-CH_2-CH_3$), 31.89 ($-O-CH_2-\underline{C}H_2-CH_2-$), 43.37 ($>\underline{C}<$), 71.62 ($-O-\underline{C}H_2-CH_2-$), 73.33 ($-\underline{C}H_2-O-CH_2-CH_2-$), 78.61 ($\geq C-\underline{C}H_2-O-\underline{C}H_2-C\leq$).

Preparation of polymer

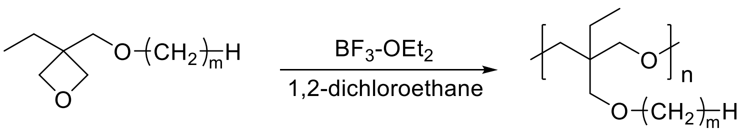

Figure S2 Polymerization reaction of CmEO (m = 1, 2, 4, 6, 8, and 12).

Each oxetane derivative was polymerized by a ring-opening cationic polymerization (ROCP) using BF_3-OEt_2 as a cationic initiator.

(**m = 1**) C1EO (1.92 mL, 0.015 mol) and the initiator (37.9 µL, 0.30 mmol) were dissolved in 1,2-dichloroethane (DCE) (total volume: 5 mL). The polymerization was performed at 0 °C for 3 h under an argon atmosphere. The reaction was quenched with the addition of aqueous 4 M NaCl/1 M NaOH (5 mL), and the reaction mixture was subsequently added to dichloromethane and washed with water. After removing dichloromethane using a rotary evaporator, the residue was dissolved in a small amount of THF. The solution was poured into a large amount of methanol to precipitate the polymerized C1EO (PC1EO). The resulting polymer was collected by decantation and dried at 130 °C under vacuum for overnight. Yield: 1.60 g, 81.9%, colorless crystal.

PC1EO ^{1}H NMR (δ, ppm from TMS in $CDCl_3$): 0.84 (t, 3H, *J* = 7.5 Hz, $-CH_2-C\underline{H}_3$), 1.38 (q, 2H, *J* = 7.3 Hz, $-C\underline{H}_2-CH_3$), 3.19 (s, 4H, $-C\underline{H}_2-O-C\underline{H}_2-$), 3.23 (s, 2H, $-C\underline{H}_2-O-C\underline{H}_3$), 3.29 (s, 3H, $-O-C\underline{H}_3$).

(**m = 2**) C2EO (3.15 mL, 0.020 mol) and the initiator (50.2 µL, 0.40 mmol) were dissolved in DCE (total volume: 5 mL). The polymerization was performed at 0 °C for 4 h under an argon atmosphere. The reaction was quenched with the addition of aqueous 4 M NaCl/1 M NaOH (5 mL), and the reaction mixture was subsequently added to chloroform and washed with water. After removing chloroform using a rotary evaporator, the residue was dissolved in a small amount of THF. The solution was poured into a large amount of methanol to precipitate the polymerized C2EO (PC2EO). The resulting polymer was collected by decantation and dried at 100 °C under vacuum for overnight. Yield: 2.06 g, 71.4%, cloudy white solid.

PC2EO ^{1}H NMR (δ, ppm from TMS in $CDCl_3$): 0.83 (t, 3H, *J* = 7.5 Hz, $\geq C-CH_2-C\underline{H}_3$), 1.15 (t, 3H, *J* = 6.8 Hz, $-O-CH_2-C\underline{H}_3$), 1.38 (q, 2H, *J* = 7.5 Hz, $\geq C-C\underline{H}_2-CH_3$), 3.20 (s, 4H, $\geq C-C\underline{H}_2-O-C\underline{H}_2-C\leq$), 3.25 (s, 2H, $-C\underline{H}_2-O-CH_2-CH_3$), 3.42 (q, 2H, *J* = 7.2 Hz, $-O-C\underline{H}_2-CH_3$).

(**m = 4**) C4EO (3.82 mL, 0.020 mol) and the initiator (50.2 µL, 0.40 mmol) were dissolved in DCE (total volume: 5 mL). The polymerization was performed at 0 °C for 3 h under argon atmosphere. The reaction was quenched with the addition of aqueous 4 M NaCl/1 M NaOH (5 mL), and the reaction mixture was subsequently added to chloroform and washed with water. After removing chloroform using a rotary evaporator, the residue was dissolved in a small amount of THF. The solution was poured into a large amount of 2-propanol to precipitate the polymerized C4EO (PC4EO). The resulting polymer was collected by decantation and dried at 130 °C under vacuum for overnight. Yield: 2.51 g, 72.9%, colorless, highly viscous liquid.

PC4EO ^{1}H NMR (δ, ppm from TMS in $CDCl_3$): 0.83 (t, 3H, J = 7.5 Hz, $\geq C-CH_2-C\underline{H}_3$), 0.91 (t, 3H, J = 7.5 Hz, $-CH_2-CH_2-C\underline{H}_3$), 1.32−1.40 (m, 4H, $\geq C-C\underline{H}_2-CH_3$, $-CH_2-C\underline{H}_2-CH_3$), 1.51 (quin., 2H, J = 7.0 Hz, $-O-CH_2-C\underline{H}_2-$), 3.19 (s, 4H, $\geq C-C\underline{H}_2-O-C\underline{H}_2-C\leq$), 3.24 (s, 2H, $-C\underline{H}_2-O-CH_2-CH_2-$), 3.35 (t, 2H, J = 6.8 Hz, $-O-C\underline{H}_2-CH_2-$).

(**m = 6**) C6EO (3.21 mL, 0.014 mol) and the initiator (40.0 μL, 0.32 mmol) were dissolved in DCE (total volume: 4.5 mL). The polymerization was performed at 0 °C for 3 h under an argon atmosphere. The reaction was quenched with the addition of aqueous 4 M NaCl/1 M NaOH (5 mL), and the reaction mixture was subsequently added to chloroform and washed with water. After removing chloroform using a rotary evaporator, the residue was dissolved in a small amount of THF. The solution was poured into a large amount of 2-propanol to precipitate the polymerized C6EO (PC6EO). The resulting polymer was collected by decantation and dried at 130 °C under vacuum for 1.5 days. Yield: 2.30 g, 80.3%, colorless, highly viscous liquid.

PC6EO ^{1}H NMR (δ, ppm from TMS in $CDCl_3$): 0.83 (t, 3H, J = 7.5 Hz, $\geq C-CH_2-C\underline{H}_3$), 0.89 (t, 3H, J = 6.5 Hz, $-CH_2-CH_2-C\underline{H}_3$), 1.26−1.33 (m, 6H, $-C\underline{H}_2-C\underline{H}_2-C\underline{H}_2-CH_3$), 1.38 (q, 2H, J = 7.3 Hz, $\geq C-C\underline{H}_2-CH_3$), 1.52 (quin., 2H, J = 6.9 Hz, $-O-CH_2-C\underline{H}_2-$), 3.19 (s, 4H, $\geq C-C\underline{H}_2-O-C\underline{H}_2-C\leq$), 3.24 (s, 2H, $-C\underline{H}_2-O-CH_2-CH_2-$), 3.34 (t, 2H, J = 6.8 Hz, $-O-C\underline{H}_2-CH_2-$).

(**m = 8**) C8EO (4.09 mL, 0.016 mol) and the initiator (40.0 μL, 0.32 mmol) were dissolved in DCE (total volume: 5.74 mL). The polymerization was performed at 0 °C for 4 h under an argon atmosphere. The reaction was quenched with the addition of aqueous 4 M NaCl/1 M NaOH (5 mL), and the reaction mixture was subsequently added to dichloromethane and washed with water. After removing dichloromethane using a rotary evaporator, the residue was dissolved in a small amount of THF. The solution was poured into a large amount of 2-propanol to precipitate the polymerized C8EO (PC8EO). The resulting polymer was collected by decantation and dried at 100 °C under vacuum for overnight. Yield: 2.33 g, 63.7%, colorless, highly viscous liquid.

PC8EO ^{1}H NMR (δ, ppm from TMS in $CDCl_3$): 0.83 (t, 3H, J = 7.5 Hz, $\geq C-CH_2-C\underline{H}_3$), 0.88 (t, 3H, J = 6.5 Hz, $-CH_2-CH_2-C\underline{H}_3$), 1.24−1.33 (m, 10H, $-C\underline{H}_2-C\underline{H}_2-C\underline{H}_2-C\underline{H}_2-C\underline{H}_2-CH_3$), 1.38 (q, 2H, J = 7.2 Hz, $\geq C-C\underline{H}_2-CH_3$), 1.52 (quin., 2H, J = 6.8 Hz, $-O-CH_2-C\underline{H}_2-$), 3.19 (s, 4H, $\geq C-C\underline{H}_2-O-C\underline{H}_2-C\leq$), 3.24 (s, 2H, $-C\underline{H}_2-O-CH_2-CH_2-$), 3.33 (t, 2H, J = 6.3 Hz, $-O-C\underline{H}_2-CH_2-$).

(**m = 12**) C12EO (5.64 mL, 0.016 mol) and the initiator (40.0 μL, 0.32 mmol) were dissolved in DCE (total volume: 5.0 mL). The polymerization was performed at 0 °C for 2 days under an argon atmosphere. The reaction was quenched with the addition of aqueous 4 M NaCl/1 M NaOH (5 mL), and the reaction mixture was subsequently added to dichloromethane and washed with water. After removing dichloromethane using a rotary evaporator, the residue was dissolved in a small amount of THF. The solution was poured into a large amount of 2-propanol to precipitate the polymerized C12EO (PC12EO). The resulting polymer was collected by decantation and dried at 100 °C under vacuum for overnight. Yield: 2.35 g, 51.6%, colorless, highly viscous liquid.

PC12EO ^{1}NMR (δ, ppm from TMS in $CDCl_3$): 0.83 (t, 3H, J = 7.3 Hz, $\geq C-CH_2-C\underline{H}_3$), 0.88 (t, 3H, J = 6.8 Hz, $-CH_2-CH_2-C\underline{H}_3$), 1.22−1.32 (m, 18H, $-C\underline{H}_2-C\underline{H}_2-C\underline{H}_2-C\underline{H}_2-C\underline{H}_2-C\underline{H}_2-C\underline{H}_2-C\underline{H}_2-C\underline{H}_2-CH_3$), 1.37 (q, 2H, J = 7.3 Hz, $\geq C-C\underline{H}_2-CH_3$),

1.52 (quin., 2H, J = 6.6 Hz, $-O-CH_2-C\underline{H}_2-$), 3.18 (s, 4H, $\geq C-C\underline{H}_2-O-C\underline{H}_2-C\leq$), 3.23 (s, 2H, $-C\underline{H}_2-O-CH_2-CH_2-$), 3.33 (t, 2H, J = 6.5 Hz, $-O-C\underline{H}_2-CH_2-$).

Molecular weight evaluation

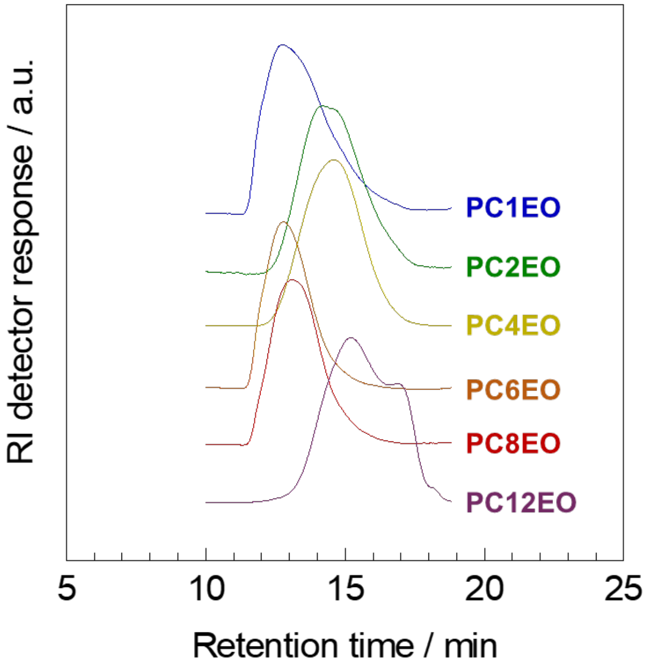

Figure S3 The chromatogram of PCmEO (m becomes longer in order from top to bottom; m = 1, 2, 4, 6, 8, and 12) obtained by gel permeation chromatography (GPC) using THF as the elution solvent, deriving polymer molecular weight. The chromatogram was detected by a refractive index (RI) detector.

Table S1 The molecular weight of PCmEO obtained from GPC chromatogram calibrated with polystyrene. M_n is the number-average molecular weight, M_w is the weight-average molecular weight, M_w/M_n is the dispersity of molecular weight, $M_{monomer}$ is monomer molecular weight, and *DP* is the degree of polymerization calculated from division of M_n by monomer molecular weight.

m	M_n / kg mol^{-1}	M_w / kg mol^{-1}	M_w/M_n	$M_{monomer}$ / g mol^{-1}	*DP*
1	59.0	147.0	2.49	130.19	453
2	26.7	48.4	1.81	144.21	185
4	27.1	47.5	1.76	172.27	157
6	101.1	172.7	1.71	200.32	505
8	73.2	135.1	1.85	228.38	321
12	11.0	23.5	2.14	284.48	38.7

Estimation of monomer density and LiTFSA concentration in polymer electrolytes

The concentration of the LiTFSA in polymer electrolytes was estimated from the reference density value for the LiTFSA melt[1] and the density of the monomer as polymer density. The monomer density was obtained from the mass ratio of each monomer and pure water with the same volume at room temperature (20–25 °C). LiTFSA concentration in polymer electrolytes was calculated assuming no density change occurs when mixing the salt and polymers. The concentration was calculated by the following formula;

$$c_{\mathrm{Li}} = \frac{(M_{\mathrm{Li}})}{\left(\frac{w_{\mathrm{Li}}}{\rho_{\mathrm{Li}}}\right) + \left(\frac{w_{\mathrm{p}}}{\rho_{\mathrm{m}}}\right)}$$

c_{Li} is LiTFSA concentration in polymer electrolytes, M_{Li} is the molar number of LiTFSA in polymer electrolytes, w_{Li} is the total weight of LiTFSA in polymer electrolytes, w_{p} is the total weight of the polymer in polymer electrolytes, ρ_{Li} is the density of the LiTFSA melt (here, 1.97 g cm^{-1}), and ρ_{m} is estimated polymer density from monomer density.

Table S2 Estimated CmEO monomer density and lithium salt concentration of polymer electrolytes.

m	monomer density / g mL^{-1}	LiTFSA ratio / wt%	LiTFSA concentration / mol L^{-1}
1	1.018	30.6	1.28
2	0.915	28.5	1.07
4	0.902	25.0	0.91
6	0.894	22.2	0.79
8	0.894	20.1	0.70
12	0.807	16.8	0.52

Electrochemical stability assessment

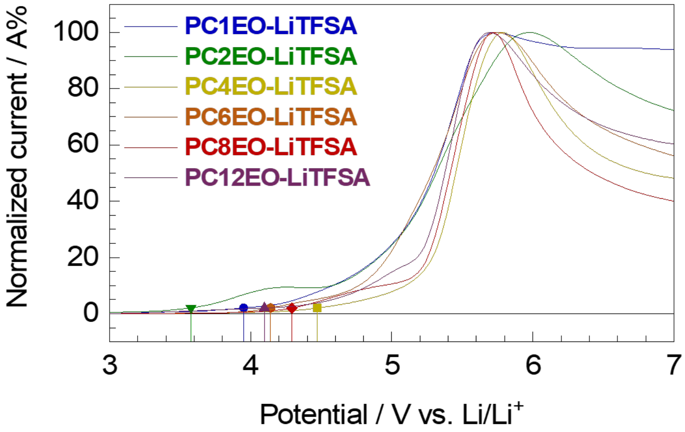

Figure S4 Linear sweep voltammogram for PCmEO-LiTFSA on stainless steel working electrode at 70 °C normalized by the peak current. Potential was scanned from the rest potential (ca. 3.0 V) to 7.0 V at a scan rate of 1 mV s^{-1}. Symbol marks indicates the starting points of current peak rising; m = 1 (blue circle), m = 2 (green down triangle), m = 4 (yellow square), m = 6 (orange hexagon), m = 8 (red diamond), and m = 12 (purple triangle).

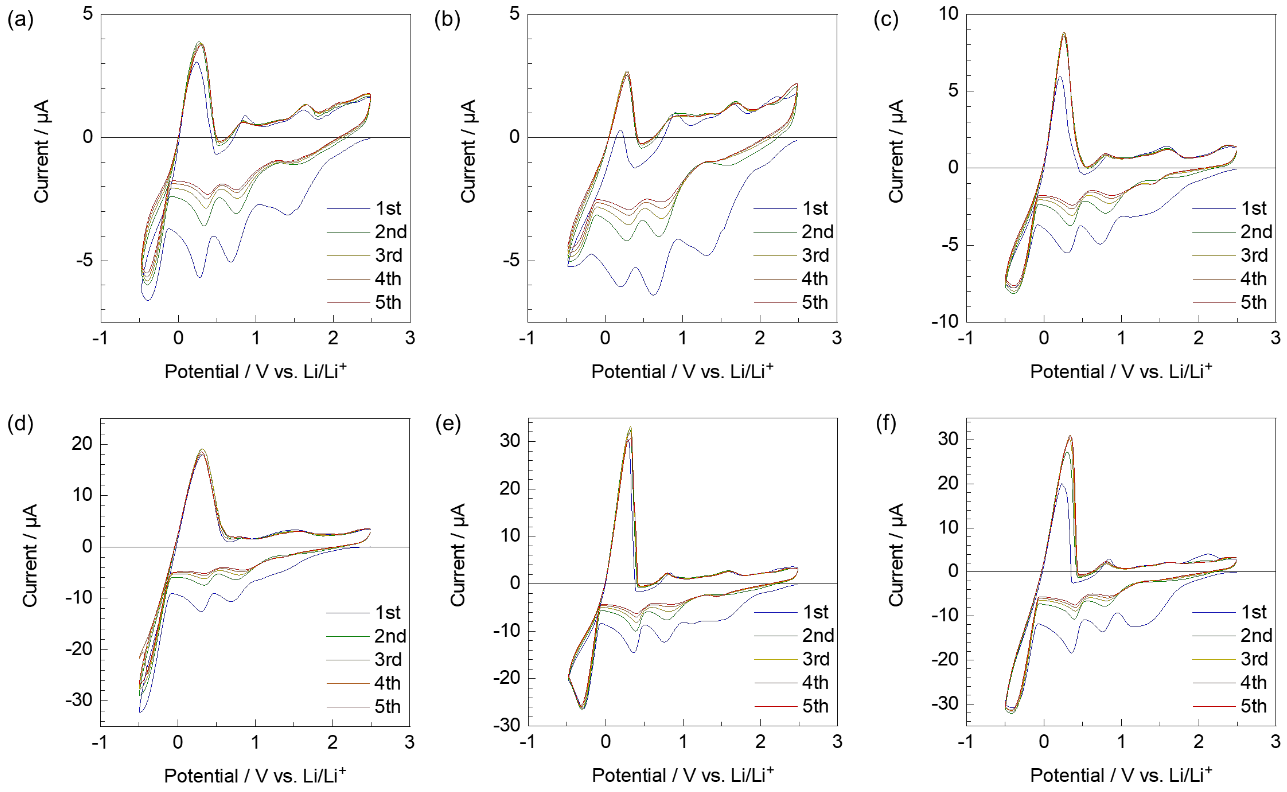

Figure S5 Cyclic voltammograms of (a) PC1EO-LiTFSA, (b) PC2EO-LiTFSA, (c) PC4EO-LiTFSA, (d) PC6EO-LiTFSA, (e) PC8EO-LiTFSA, and (f) PC12EO-LiTFSA on Ni electrode at a scan rate of 1 mV s^{-1} at 70 °C.

Gas chromatography-mass spectrometry (GC-MS)

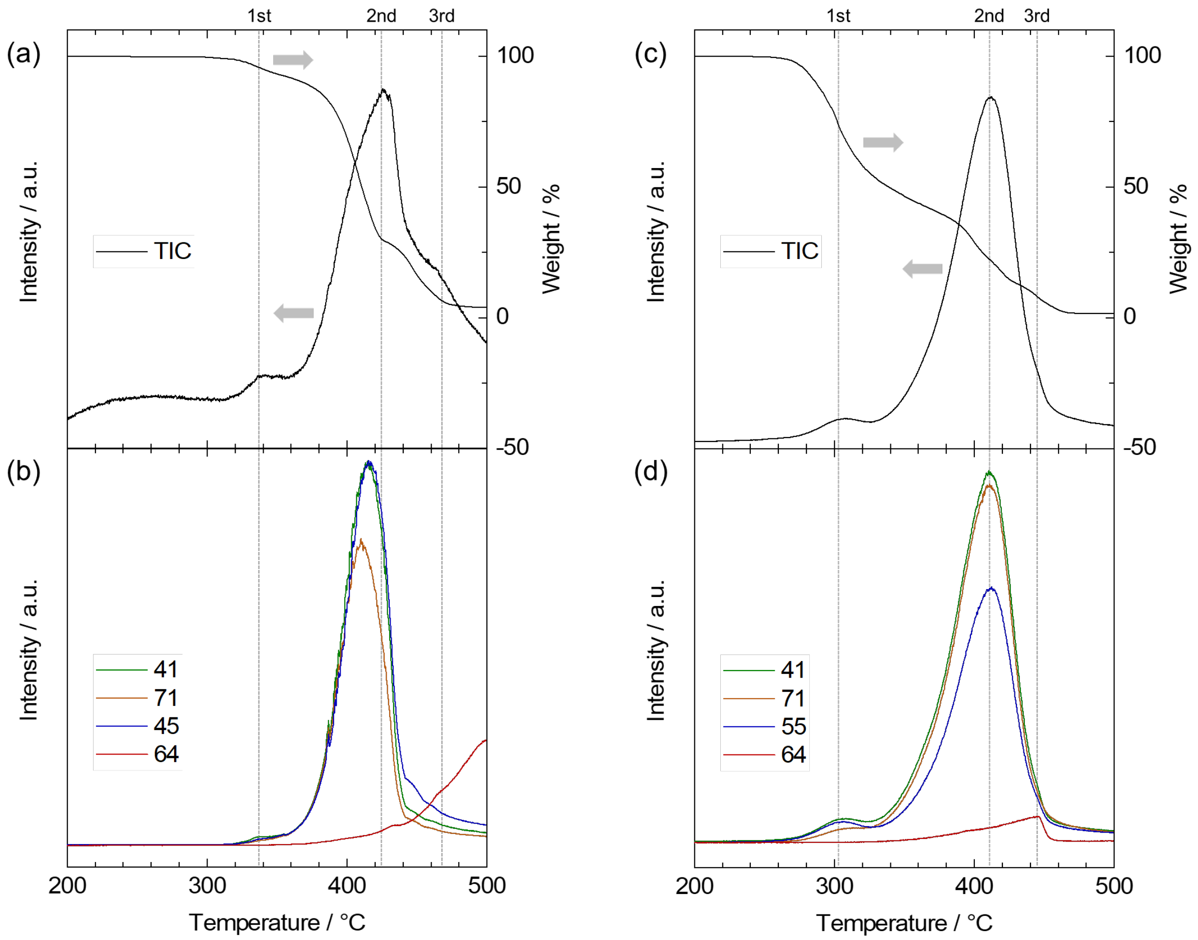

Figure S6 Gas chromatography-mass spectrometry (GC-MS) curves for (a), (b) PC1EO-LiTFSA and (c), (d) PC12EO-LiTFSA, recorded from 150 °C to 500 °C under He flow. TIC in (a) and (c) indicates the total ion chromatogram. Assignment for each m/z is as follows. (b) m/z = 41: $^{\bullet}CH_2{-}CH{=}CH_2$ derived from the decomposition of ethyl group, m/z = 71: $O{=}CH{-}^{\bullet}CH{-}CH{=}O$ derived from the decomposition of polymer main chain, m/z = 45: $^{\bullet}CH_2{-}O{-}CH_3$ derived from the decomposition of methoxymethyl group (polymer side chain), and m/z = 64: $O{=}S{=}O$ derived from the decomposition of TFSA anion. (d) m/z = 41: $^{\bullet}CH_2{-}CH{=}CH_2$ derived from the decomposition of ethyl group or dodecyl group, m/z = 71: $O{=}CH{-}^{\bullet}CH{-}CH{=}O$ derived from the decomposition of polymer main chain, m/z = 55: $CH_3{-}^{\bullet}CH{-}CH{=}CH_2$ derived from the decomposition of dodecyl group, and m/z = 64: $O{=}S{=}O$ derived from the decomposition of TFSA anion.

Thermal transition behavior of Polymer electrolytes and pure polymers obtained from DSC curves

Table S3 Thermal transition temperature of the pure PCmEO and the corresponding polymer electrolytes (PEs), obtained from DSC curves; T_g is glass transition temperature determined as the intersection of the baseline and the diagonal line of the baseline shift, T_m is the melting temperature determined as the intersection of the baseline and the diagonal line of the endothermic peak, ΔH_m is the enthalpy derived from the melting of the crystalline phase determined as the area of the endothermic peak, T_c is the re-crystallization temperature determined as the intersection of the baseline and the diagonal line of exothermic peak, and ΔH_c is the enthalpy derived from the formation of the crystalline phase determined as the area of the exothermic peak.

m		T_g / °C	T_m / °C	ΔH_m / J g^{-1}	T_c / °C	ΔH_c / J g^{-1}
1	Pure	−34.7	64.4	36.6	–	–
	PE	−16.6	–	–	–	–
2	Pure	−41.9	27.6	12.8	2.6	13.1
	PE	−33.1	–	–	–	–
4	Pure	−63.5	–	–	–	–
	PE	−50.4	–	–	–	–
6	Pure	−70.3	–	–	–	–
	PE	−57.2	–	–	–	–
8	Pure	−72.8	−24.7	7.2	−49.5	7.5
	PE	−68.3	–	–	–	–
12	Pure	−100.9	−31.1	67.3	–	–
	PE	−95.3	−34.8	41.3	–	–

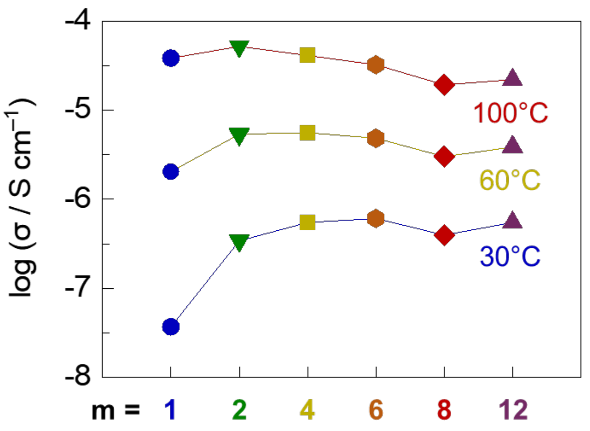

Figure S7 Alkyl side chain length dependence of the ionic conductivity (σ) for PCmEO-LiTFSA at 30 °C, 60 °C, and 100 °C. Each symbol shows m = 1 (blue circle), m = 2 (green down triangle), m = 4 (yellow square), m = 6 (orange hexagon), m = 8 (red diamond), and m = 12 (purple triangle).

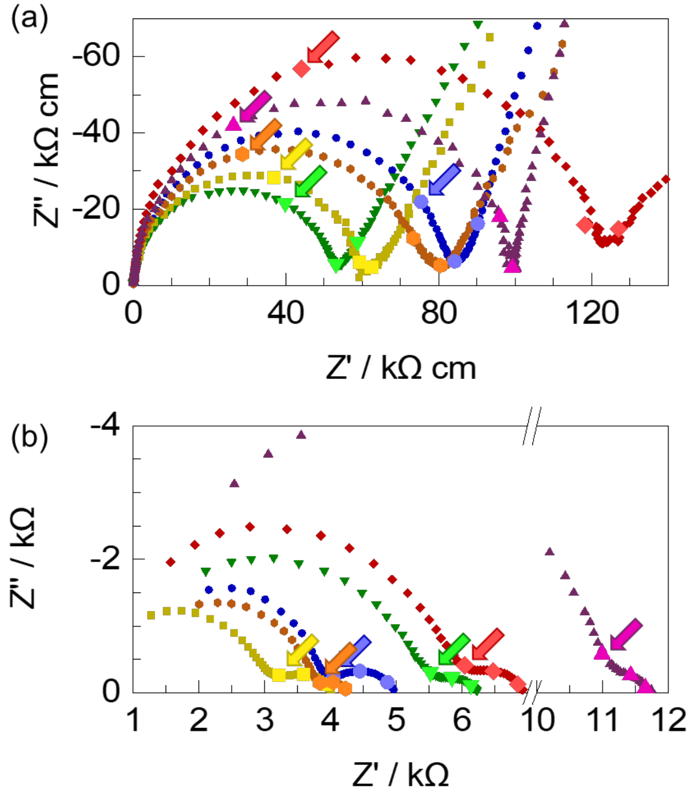

Figure S8 Nyquist plot for the electrochemical impedance spectroscopy (EIS) at 80 °C using the symmetric cell with (a) stainless-steel blocking electrode for ionic conductivity measurements and (b) lithium non-blocking electrode for analysis of lithium-ion transfer at electrode-electrolyte interface. Three large and bright marks indicate 10000 (indicated with allow), 1000, and 100 Hz frequency response.

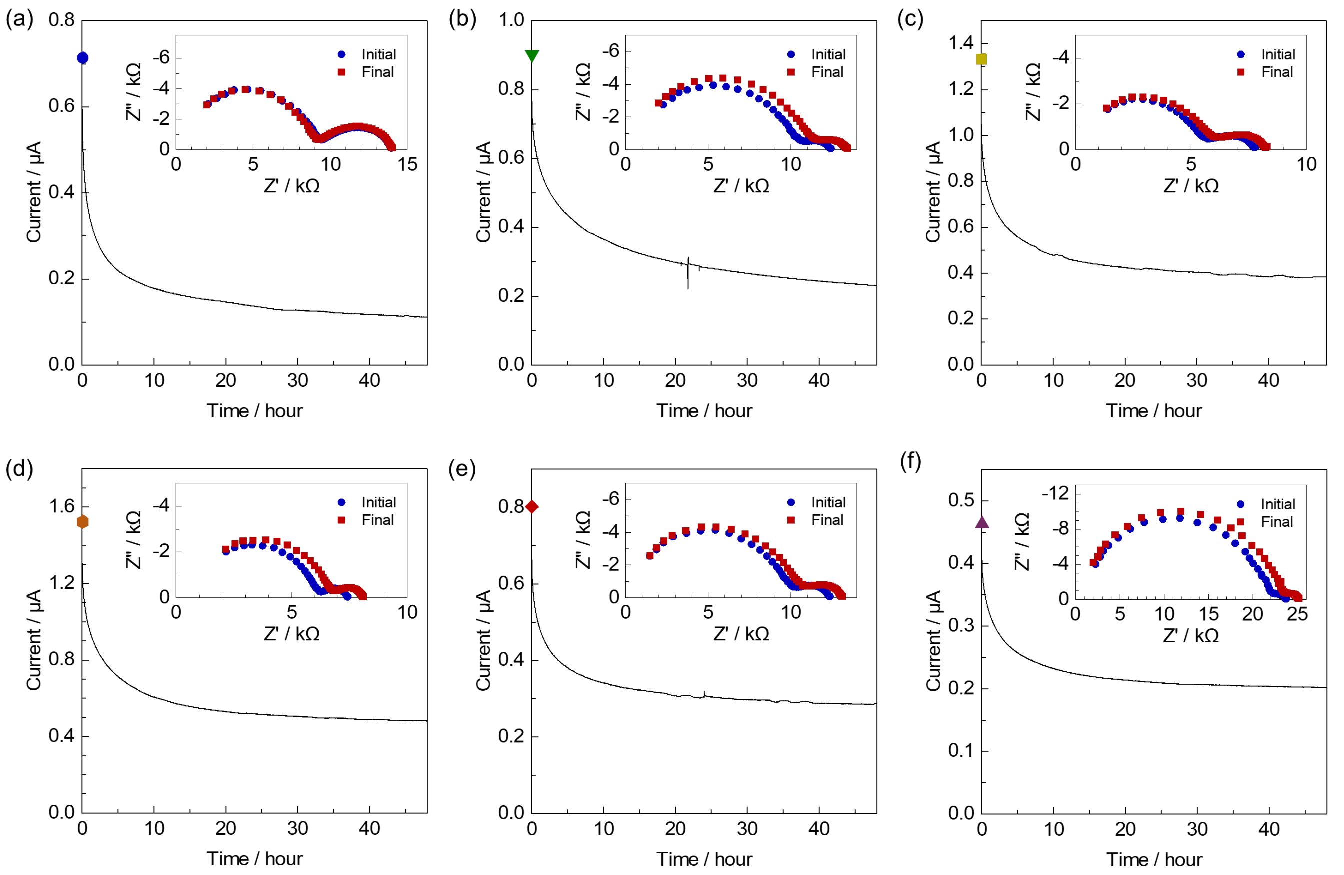

Figure S9 Chronoamperogram at 10 mV at 70 °C and corresponding Nyquist plot for the electrochemical impedance spectroscopy (EIS) before and after the chronoamperometry; (a) m = 1, (b) m = 2, (c) m = 4, (d) m = 6, (e) m = 8, and (f) m = 12.

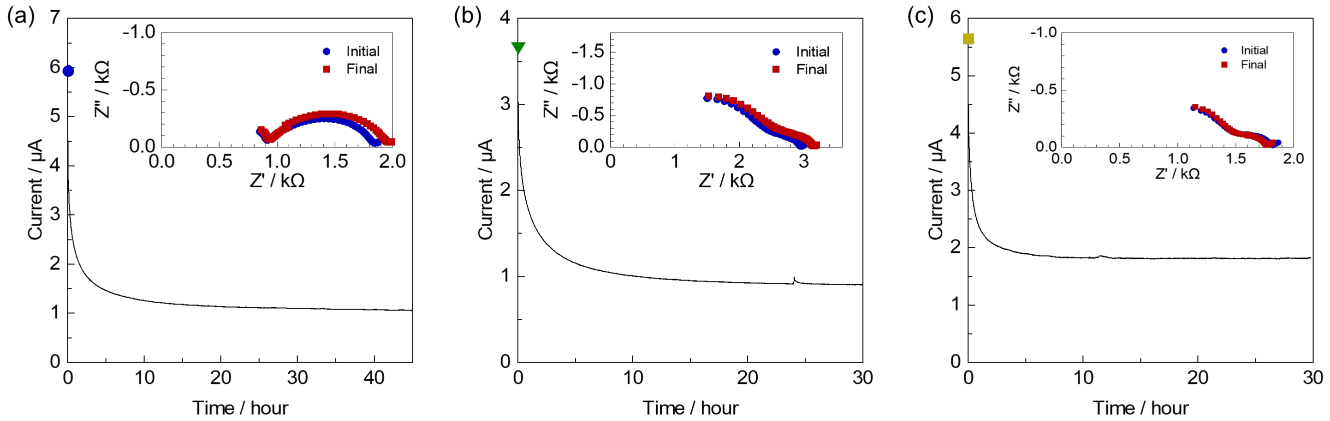

Figure S10 Chronoamperogram at 10 mV at 100 °C and corresponding Nyquist plot for the electrochemical impedance spectroscopy (EIS) before and after the chronoamperometry; (a) m = 1, (b) m = 2, (c) m = 4, (d) m = 6, (e) m = 8, and (f) m = 12.

Table S4 Electrochemical parameters obtained from Figures S9 and S10, where I_0 is the initial current of the potentiostatic polarization, I_{SS} is the steady-state current of the polarization, $R_B{}^i$ is the bulk resistance before the polarization, and $R_B{}^f$ is the steady-state bulk resistance during the polarization, $R_I{}^i$ is the interfacial resistance before the polarization, $R_I{}^f$ is the steady-state interfacial resistance during the polarization, and calculated Li^+ transference number (t_{Li+}).

Temp. / °C	m	I_0 / μA	I_{SS} / μA	$R_B{}^i$ / kΩ	$R_B{}^f$ / kΩ	$R_I{}^i$ / kΩ	$R_I{}^f$ / kΩ	t_{Li+}
70	1	0.716	0.111	9.38	9.25	4.17	4.32	0.15
	2	0.903	0.231	10.20	11.03	1.75	1.78	0.28
	4	1.337	0.380	5.65	6.01	1.92	2.06	0.30
	6	1.531	0.483	6.25	6.82	1.06	1.17	0.34
	8	0.803	0.285	10.00	10.51	2.15	2.28	0.37
	12	0.465	0.201	21.86	23.10	1.91	1.96	0.46
100	1	5.945	1.056	0.915	0.933	0.805	0.947	0.18
	2	3.681	0.903	2.420	2.530	0.470	0.479	0.26
	4	5.655	1.817	1.438	1.452	0.364	0.328	0.32

IR spectra

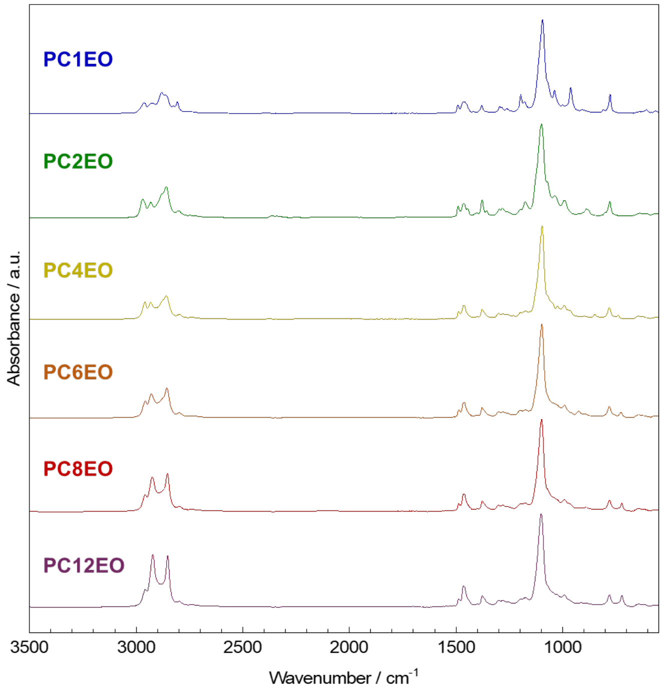

Figure S11 Attenuated total reflection (ATR) infrared (IR) spectra of the PCmEO (m becomes larger in order from top to bottom; m = 1, 2, 4, 6, 8, and 12).

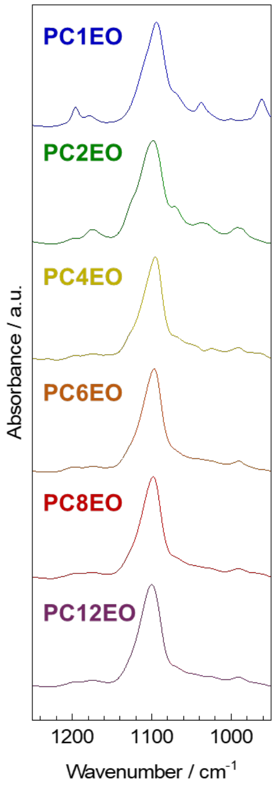

Figure S12 Attenuated total reflection (ATR) infrared (IR) spectra of the ν_{as}(COC) region for PCmEO (m becomes larger in order from top to bottom; m = 1, 2, 4, 6, 8, and 12).

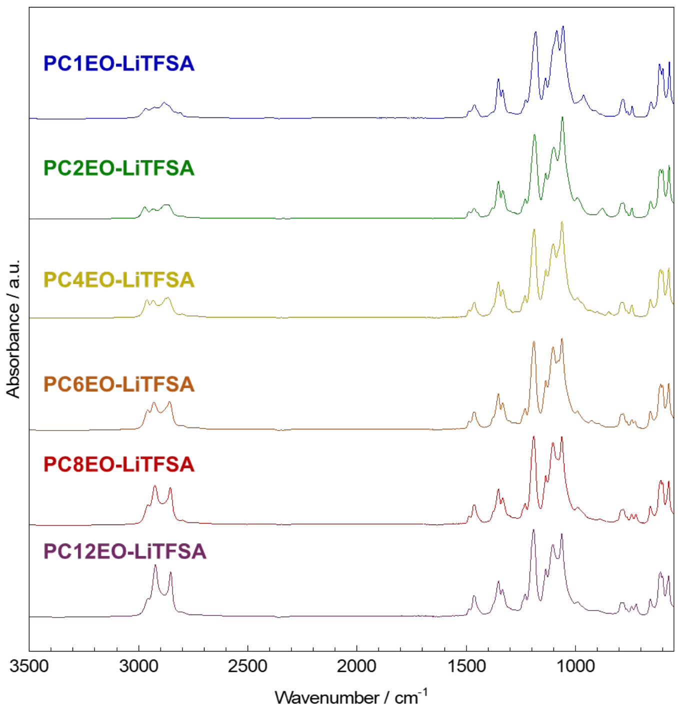

Figure S13 Attenuated total reflection (ATR) infrared (IR) spectra of the PCmEO-LiTFSA (m becomes larger in order from top to bottom; m = 1, 2, 4, 6, 8, and 12).

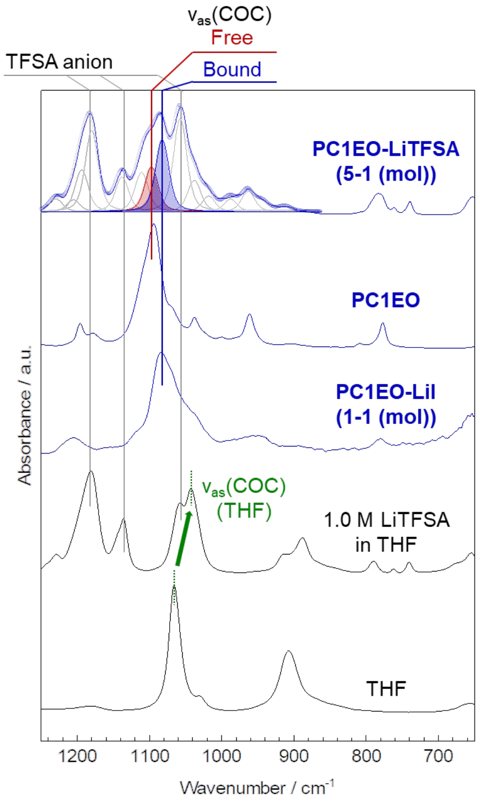

Figure S14 Deconvoluted ATR-IR spectra of the ν_{as}(COC) region for PC1EO-LiTFSA. ATR-IR spectra for pure PC1EO, PC1EO-LiI (1 polymer unit mol / 1 LiI mol), 1.0 M LiTFSA in THF, and pure THF is also shown for comparison.

Table S5 Assignment of the infrared (IR) peaks for PCmEO (m =1, 4, and 12).

Assignment[2]	Melting PEO[2]	PEO in H_2O	PC1EO	PC4EO	PC12EO
$\nu_{as}(CH_3)$			2963 (11)	2959 (18)	2958 (18)
$\nu_{as}(CH_2)$		2914 (16)	2927 (11)	2932 (18)	2922 (56)
$\nu_s(CH_2)$		2880 (17)	2880 (22)	2874 (21)	
			2862 (19)	2859 (25)	2852 (55)
$\nu_s(CH_3)$			2825 (8)		
$\nu_s(CH_2)$			2807 (12)	2797 (5)	2798 (6)
$\delta_s(CH_3)$			1491 (9)	1487 (8)	1488 (9)
$\delta_s(CH_2)$	1460 m	1470 (15)	1457 (13)	1462 (15)	1465 (22)
		1455 (15)			
$\gamma_s(CH_2)$	1352 m	1349 (24)	1379 (8)	1377 (10)	1377 (12)
	1326 w	1332 (11)			
$\gamma_{as}(CH_2)$	1296 m	1302 (16)	1295 (7)	1300 (6)	1300 (7)
		1288 (17)	1286 (7)	1280 (6)	1280 (7)
	1249 m	1251 (19)	1261 (5)	1257 (5)	1255 (5)
				1231 (4)	
$\nu(COC)$			1196 (20)	1198 (7)	1197 (8)
			1178 (12)	1174 (9)	1174 (10)
$\nu_{as}(COC)$, $\nu(CC)$, $\delta_{as}(CH_2)$	1140 sh	1135 (41)			
	1107 s	1082 (100)	1094 (100)	1096 (100)	1100 (100)
			1070 (34)	1069 (25)	1069 (23)
	1038 m	1039 (34)	1038 (25)	1047 (17)	1046 (15)
				1025 (14)	1029 (13)
$\nu(COC)$, $\nu(CC)$	992 w	995 (18)	1000 (9)	991 (14)	990 (13)
$\nu(COC)$, $\delta_{as}(CH_2)$	945 m	948 (42)	961 (28)	965 (10)	
	915 sh	913 (25)			
		884 (23)			
	855 m	835 (42)			
$\delta_{as}(CH_2)$, $\gamma_{as}(CH_2)$	810 sh		809 (4)	848 (5)	
			777 (21)	780 (12)	780 (12)
				739 (4)	721 (9)

The numbers in parentheses show the relative height of each peak obtained from the observed IR spectra.

Table S6 Assignment of the infrared (IR) peak for LiTFSA.

Assignment[3]	LiTFSA in PEO[3]	LiTFSA in THF	LiTFSA in PC1EO
$\nu_{as}^{(i.p.)}$ (SO_2)	1354 (76)	1354 (39)	1352 (45)
$\nu_{as}^{(o.p.)}$ (SO_2)	1334 (48)	1333 (29)	1333 (32)
$\nu_s(CF_3)$	1240 (3)	1243 (11)	1242 (10)
$\nu_{as}(CF_3)$	1227 (45)	1229 (20)	1228 (21)
	1195 (100)	1181 (100)	1182 (100)
$\nu_s^{(o.p.)}$ (SO_2)	1136 (71)	1136 (54)	1136 (47)
ν_{as}(SNS)	1060 (71)	1058 (69*)	1056 (107*)
ν(CS)	788 (11)	790 (14)	784 (22*)
ν_s(SNS)	761 (5)	762 (6)	761 (8)
$\delta_s(CF_3)$	739 (9)	741 (13)	740 (14)
δ(SNS)	655 (11)	655 (22)	653 (19)
$\delta_{as}^{(o.p.)}$ (SO_2)	618 (43)	614 (60)	615 (63)
$\delta_{as}^{(i.p.)}$ (SO_2)	604 (26)	600 (58)	600 (58)
$\delta_{as}(CF_3)$	571 (27)	570 (65)	570 (65)

The numbers in parentheses show the relative height of each peak obtained from the observed IR spectra except for reference value of LiTFSA in PEO.

* Peak overlaps polymer peak

REFERENCES

(1) Kubota, K.; Tamaki, K.; Nohira, T.; Goto, T.; Hagiwara, R. Electrochemical Properties of Alkali Bis(Trifluoromethylsulfonyl)Amides and Their Eutectic Mixtures. *Electrochim. Acta* **2010**, *55* (3), 1113–1119. https://doi.org/10.1016/j.electacta.2009.09.024.

(2) Matsuura, H.; Fukuhara, K. Vibrational Spectroscopic Studies of Conformation of Poly(Oxyethylene). II. Conformation–Spectrum Correlations. *J. Polym. Sci. Part B Polym. Phys.* **1986**, *24* (7), 1383–1400. https://doi.org/10.1002/polb.1986.090240702.

(3) Rey, I.; Johansson, P.; Lindgren, J.; Lassègues, J. C.; Grondin, J.; Servant, L. Spectroscopic and Theoretical Study of (CF3SO2)2N- (TFSI-) and (CF3SO2)2NH (HTFSI). *J. Phys. Chem. A* **1998**, *102* (19), 3249–3258. https://doi.org/10.1021/jp980375v.
